# 3D Reconstruction and Standardization of the Rat Vibrissal Cortex for Precise Registration of Single Neuron Morphology

**DOI:** 10.1371/journal.pcbi.1002837

**Published:** 2012-12-20

**Authors:** Robert Egger, Rajeevan T. Narayanan, Moritz Helmstaedter, Christiaan P. J. de Kock, Marcel Oberlaender

**Affiliations:** 1 Digital Neuroanatomy, Max Planck Florida Institute, Jupiter, Florida, United States of America; 2 Center for Neurogenomics and Cognitive Research, Neuroscience Campus Amsterdam, VU University Amsterdam, Amsterdam, the Netherlands; 3 Structure of Neocortical Circuits Group, Max Planck Institute of Neurobiology, Martinsried, Germany; NIH, United States of America

## Abstract

The three-dimensional (3D) structure of neural circuits is commonly studied by reconstructing individual or small groups of neurons in separate preparations. Investigation of structural organization principles or quantification of dendritic and axonal innervation thus requires integration of many reconstructed morphologies into a common reference frame. Here we present a standardized 3D model of the rat vibrissal cortex and introduce an automated registration tool that allows for precise placement of single neuron reconstructions. We (1) developed an automated image processing pipeline to reconstruct 3D anatomical landmarks, i.e., the barrels in Layer 4, the pia and white matter surfaces and the blood vessel pattern from high-resolution images, (2) quantified these landmarks in 12 different rats, (3) generated an average 3D model of the vibrissal cortex and (4) used rigid transformations and stepwise linear scaling to register 94 neuron morphologies, reconstructed from *in vivo* stainings, to the standardized cortex model. We find that anatomical landmarks vary substantially across the vibrissal cortex within an individual rat. In contrast, the 3D layout of the entire vibrissal cortex remains remarkably preserved across animals. This allows for precise registration of individual neuron reconstructions with approximately 30 µm accuracy. Our approach could be used to reconstruct and standardize other anatomically defined brain areas and may ultimately lead to a precise digital reference atlas of the rat brain.

## Author Summary

For studying the neural basis of perception and behavior, it would be ideal to directly monitor sensory-evoked excitation streams within neural circuits, at sub-cellular and millisecond resolution. To do so, reverse engineering approaches of reconstructing circuit anatomy and synaptic wiring have been suggested. The resulting anatomically realistic models may then allow for computer simulations (*in silico* experiments) of circuit function. A natural starting point for reconstructing neural circuits is a cortical column, which is thought to be an elementary functional unit of sensory cortices. In the vibrissal area of rodent somatosensory cortex, a cytoarchitectonic equivalent, designated as a ‘barrel column’, has been described. By reconstructing the 3D geometry of almost 1,000 barrel columns, we show that the somatotopic layout of the vibrissal cortex is highly preserved across animals. This allows generating a standard cortex and registering neuron morphologies, obtained from different experiments, to their ‘true’ location. Marking a crucial step towards reverse engineering of cortical circuits, the present study will allow estimating synaptic connectivity within an entire cortical area by structural overlap of registered axons and dendrites.

## Introduction

The morphology of neurons has been of interest for three main reasons. First, the morphology of the soma, dendrites, and axon is commonly used to identify types of neurons [1], [2], [3], [4], [5]. Secondly, the detailed morphology of soma and dendrites has been analyzed with respect to their biophysical effect on electrical properties [6], [7], [8], [9]. Thirdly, the morphology of axons and dendrites has been used to infer synaptic connectivity between neurons by structural overlap [3], [4], [10], [11], [12], [13].

For classification of neuronal cell types and analyzing biophysical properties, a single neuron may be a sufficient reference frame for morphological analysis. In contrast, the aim of inferring synaptic connectivity from structural overlap of neuronal morphologies requires the placement of neurons into a reference frame that is sufficiently precise and invariant to variability between experiments. Quantitative registration methods have been applied to neurons in the mammalian cortex from *in vitro* preparations with two-dimensional (2D) registration [14], [15]. Three-dimensional (3D) registration approaches were so far limited to various insect model systems, such as the bee and fly brains [16], [17]. In contrast to the stereotypic layout of these insect brains [18], where the number of neurons and even the neuronal projection patterns are often preserved across animals [19], [20], the mammalian cortex is likely to be more heterogeneous and variable across animals.

For the analysis of cortical neuron ensembles in 3D, for example from experiments carried out *in vivo* (relieving the restriction to a tissue slice), a 3D registration is required, especially if neurons from many different experiments are to be combined. We therefore developed a set of tools that allow (i) reconstructing anatomical landmarks with 1 µm resolution, (ii) generating a standardized average 3D cortex model and (iii) precise registration of 3D neuron morphologies, obtained from *in vivo* preparations.

Due to its well-defined structural and functional layout, subdivided vertically into cortical barrel columns and horizontally into six cortical layers (L1–6), the rodent vibrissal cortex is a natural starting point for generating a precise 3D anatomical model of the mammalian cortex. A cortical column is thought to be the elementary functional unit of sensory cortices [21], [22]. The barrel columns in the vibrissal area of rodent somatosensory cortex (S1) are regarded as cytoarchitectonic equivalents of these functional columns [23], [24]. In the present study, we define the dimensions of barrel columns by staining for Cytochrome-oxidase in L4 and extrapolating the circumference of the respective L4 barrels along their vertical axes towards the pia and white matter. This cylindrical approximation renders one way to describe the 3D extent of cortical barrel columns, but alternative definitions, for example, based on thalamocortical projections [25], dendrite innervations [3] or intracortical connectivity patterns [26], [27] exist.

Despite multiple studies that investigated the geometry of the rodent vibrissal cortex [25], [28], [29], [30], a quantitative 3D description of the variability of barrel column dimensions and orientations within the vibrissal cortex and across animals is lacking. Here we developed criteria to automatically extract the dimensions of the barrels, the pia and white matter surfaces and the orientation of the barrels and respective cortical columns with high precision. We find that the variability of individual anatomical parameters is surprisingly small across different animals. In contrast, the parameters within individual animals differ substantially.

The large anatomical variability within the vibrissal cortex of individual animals demands that neuron reconstructions need to be registered as close as possible to their original location. The automated registration tool presented here meets this demand. Rigid transformations and stepwise linear scaling along the vertical column axis are used to match the reference landmarks of a reconstructed neuron to their respective counterparts in the standardized cortex model. The 3D reconstructions of somata, dendrites and axons from *in vivo* preparations can thus be placed at their true cortical location with a precision of approximately 30 µm.

## Results

### Reconstruction of anatomical landmarks in rat vibrissal cortex

**Figure 1:**
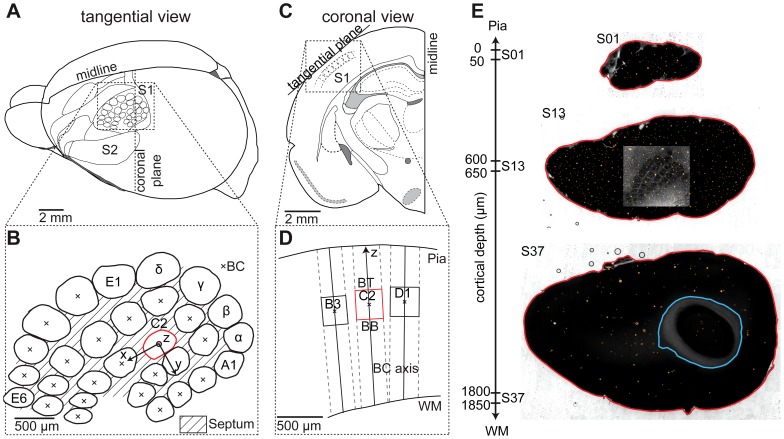
3D parameterization of barrels and barrel columns in rat vibrissal cortex. (A) Tangential view of the left hemisphere of a rat brain. The barrel field is located in the primary somatosensory cortex (S1), adjacent to the secondary somatosensory cortex (S2). (B) The barrels are arranged in a somatotopic layout of rows (A–E) and arcs (1–6). The four barrels in front of the first arc are given greek labels (α-δ). The barrel center (BC) is the centroid of a barrel and is used to describe the 3D location of individual barrels. The coordinate system used to describe the 3D layout of the barrel field based on the position of the BCs is centered on the C2 barrel (red), which is centrally located within the barrel field. The z axis points vertically along the C2 barrel column axis, the x axis is chosen to point towards the C3 barrel center (approximately along the row) and the y axis is perpendicular to the x and z axes and points approximately along the arc. (C) View of a coronal section of the left hemisphere (see dashed line in a). Barrels can be visualized by preparing cortical sections tangential to the barrel cortex. (D) The barrel cortex is organized into vertical barrel columns. These are obtained by cylindrical extrapolation of the barrel outlines along their respective BC axis to the pia and subcortical White matter (WM), respectively. The location of a barrel along the BC axis is described by the barrel top (BT) and barrel bottom (BB) points. (E) Tangential sections through rat cortex, indicating the relative depth below the pia, with automatically detected anatomical landmarks: red – pia, blue – WM, orange – blood vessels. The inset in section S13 shows an example of a high-resolution optical section of the barrel field.

The vibrissal cortex in rats comprises 30 large barrels in L4, separated by septa between them (**Figure 1A**). The layout of the barrel field, as revealed by Cytochrome-oxidase staining [31], resembles the layout of the large facial whiskers on the animal's snout, which are organized into rows (A–E) and arcs (α-δ, A1–4, B1–4, C/D/E1–6) (**Figure 1B**).

Taking a coronal section through the barrel field reveals that the curvatures of the pia and white matter (WM) surfaces (**Figure 1C**) differ across the vibrissal cortex. This results in location-specific cortical thickness and barrel depth, as well as tilted orientations of the vertical barrel column axes with respect to each other (**Figure 1D**).

We defined five parameters for each barrel column to describe this location-specific 3D layout of the vibrissal cortex: (i) the *barrel area*, defined as the maximal circumference of the L4 barrel in the tangential plane (**Figure 1B**), (ii) the *barrel top (BT)*, defined as the closest point of the barrel to the pia in the coronal plane (**Figure 1D**), (iii) the *barrel bottom (BB)*, defined as the closest point of the barrel to the WM. Together, BB and BT define the vertical extent (i.e., height) of a barrel. The maximal barrel circumference and the height yield the definition of its centroid (i.e., barrel center, BC). The two remaining parameters are (iv) the *barrel column orientation (BC axis)*, defined as the shortest perpendicular axis from the BC to the pia above the barrel (**Figure 1D**) and (v) the *barrel column height*, defined by extrapolating the barrel circumference along the BC axis towards the WM and pia, respectively (i.e., pia-WM distance).

We determined these five parameters for 984 barrels from 104 different rats. To do so, brains were cut approximately tangential to the barrel field into 50 or 100 µm thick vibratome sections (**Figure 1E**). Ranging from the pia to the WM, the resulting 24 or 48 brain sections (S01–S48) were stained for Cytochrome-oxidase to reveal the barrel field in L4 (e.g., S13 in **Figure 1E**).

We manually traced 637 individual barrels on low-resolution images from 100 µm thick sections in 92 rats using Neurolucida software (MicroBrightfield, Williston, VT, USA). Only clearly stained barrels were traced, one contour per brain section. In addition, pia and WM contours were traced for all sections. The resultant average barrel area was 9.8±1.9×10^4^ µm^2^ (mean ± SD). The average barrel height was 299±92 µm. The average pia-WM distance was 1949±100 µm.

**Figure 2:**
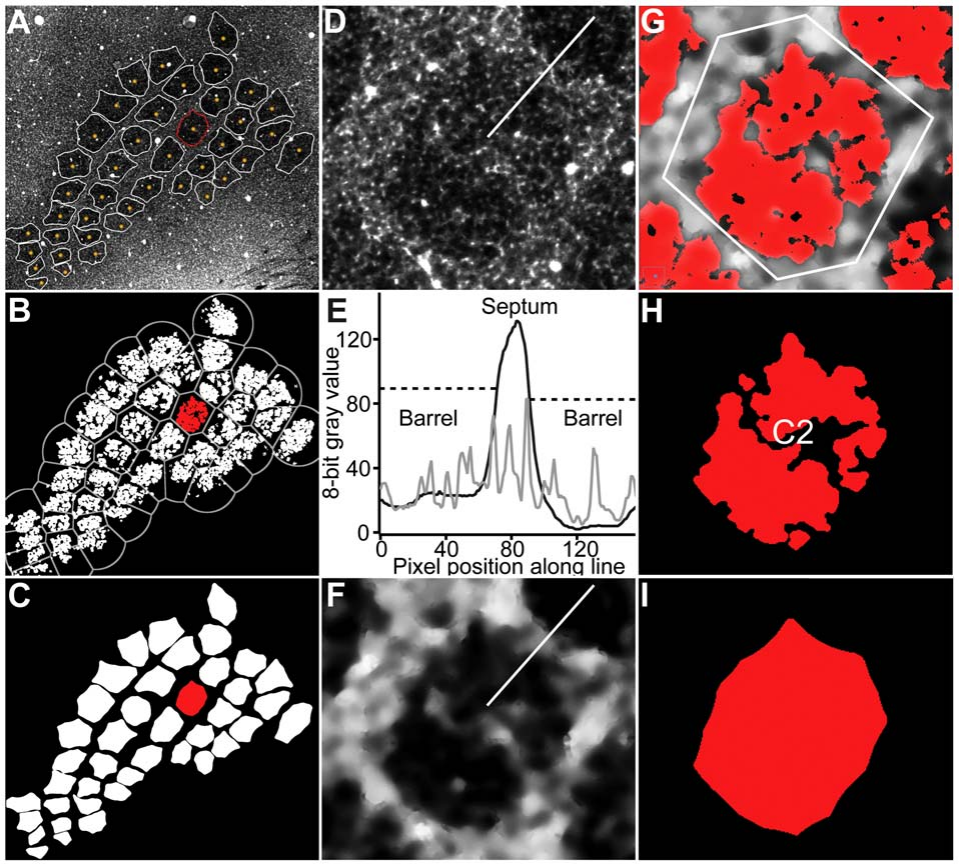
Reconstruction of barrel outlines from high-resolution optical section. (A) Optical section of the barrel field. Manual landmarks (yellow) are placed in barrels that are going to be segmented. White contours show the final segmentation result of this section. (B) Result of gray-value based image segmentation. Gray lines overlaid indicate the Voronoi regions (VR) of the manual landmarks used for region growing (G). (C) Final result of VR-based barrel segmentation. (D) Raw image data of barrel with red contour in A. Line shows pixels included in the line profile in E. (E) Line profiles of the intensity values along the lines before (gray; see D) and after filtering (black; see F). Dashed lines indicate threshold values used to separate different barrels from septum. (F) Result of image filtering. Line shows pixels included in the line profile in E. (G) VR-based region growing. White lines indicate border of the VR of the manual landmark. (H) VR-based barrel detection turns the binary- segmented image into an object image by assigning every segmented pixel to a barrel. (I) Closing merges all fragments belonging to one barrel.

**Figure 3:**
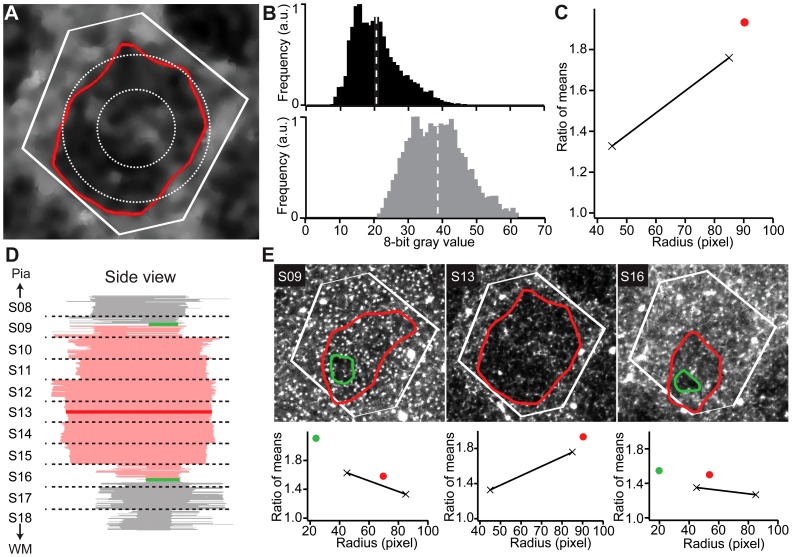
Reconstruction of 3D barrel dimensions. (A) Barrel contour in a single optical section overlaid on the filtered image (gray values linearly enhanced for visualization). White lines indicate border of the VR of this barrel. Dashed circles with diameters of 90 and 170 pixels (approx. 165 and 315 µm) indicate the regions used to estimate the barrel extent. (B) Histogram of background pixels inside the barrel contour in A (black) and histogram of background pixels in septum (gray), i.e. outside of the barrel contour and inside of the VR. Dashed white lines mark mean gray value. (C) Ratio of means inside/outside of the contour as a function of the contour radius. Black: circular regions in A; red: segmented contour. (D) Side view of all segmented contours. Subsequent tangential sections are aligned using blood vessels. (E) Top row: Individual optical sections from different tangential sections (marked bold in D). Red – regular segmented contours. Green – optimized minimal contours. Bottom row: Radial dependence of the ratio of means for all contours in the corresponding optical sections. The slope of the black line gives an estimate of the relative size of the true barrel extent compared to the segmented contour. At the top and bottom of the barrel it may be necessary to segment an optimized minimal contour (see Supplemental Materials).

The manual determination of the vertical extent of the barrel (i.e., BT and BB) proved to be difficult, because barrels were tilted with respect to the vertical cortex axis within a brain section. Consequently, we decided to determine the barrel dimensions by more objective criteria. Using an automated image processing pipeline (**Figure S1, S2, S3**) and high-resolution image stacks, the contrast between barrels and the septum was enhanced (**Figure 2**). This allowed determining the BT and BB as local minima in diameter of the extracted barrel contours (**Figure 3**).

Using this automated tracing method, we reconstructed 347 barrels from 50 µm thick sections in 12 different rats (6 male, 6 female). The average area of the automatically extracted barrels was 9.9±1.7×10^4^ µm^2^. The average pia-WM distance was 1929±99 µm. Because the mean values as well as the standard deviations (SDs) of the two parameters were identical to their manually determined counterparts, we regard our automated algorithms as sufficiently accurate to reconstruct the five anatomical parameters describing the barrel field.

The automatically determined barrel height (348±34 µm) was slightly different from its manual counterpart (299±92 µm). Given the difficulties in manually determining the vertical borders of the barrels, we regard the automated result as more accurate. Further, we determined a systematic error of ∼10 µm for the automated detection of BT and BB, respectively. Thus, the automatically determined SD of 34 µm in barrel height likely reflects the ‘true’ biological variability between animals. In contrast, the 3-fold larger manually determined SD in barrel height of 92 µm may primarily reflect systematic limitations of the manual tracings and hence conceals the biological variability.

Consequently, the automated pipeline of imaging and image processing, presented here, is a fast and precise alternative to extract the 3D geometry of the vibrissal cortex, reaching at least the same accuracy as manual tracings, by using a smaller sample size. Therefore, only the 12 automatically reconstructed vibrissal cortices were subsequently used for quantification and standardization of the five geometrical parameters.

### Anatomical landmarks vary within rat vibrissal cortex

The cortical column and its cytoarchitectonic equivalent in the vibrissal cortex, the barrel column, has been regarded as an elementary building block of sensory cortices [32], [33]. Accordingly, assuming a stereotypic column layout throughout the cortex, average dimensions were used to describe the 3D column dimensions. Here, we determined an average barrel area and height of ∼100,000 µm^2^ and 300 µm, respectively, yielding a barrel volume of ∼0.03 mm^3^. Combined with an average column height of ∼2,000 µm, we obtained an average barrel column volume of 0.2 mm^3^. These values were in good agreement with previous 2D measurements [25].

**Figure 4:**
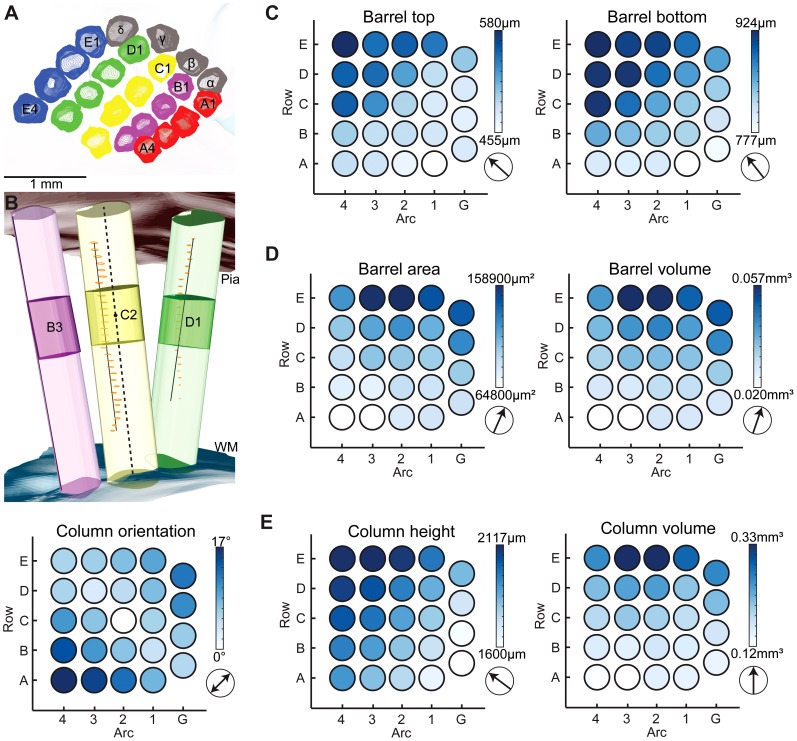
3D reconstruction of barrels and barrel columns. (A) Segmented barrel contours are smoothed in the z-direction to remove segmentation artifacts. (B) Anatomical structures are reconstructed in 3D. Blood vessels (orange) are reconstructed as 3D lines. Pia and WM are reconstructed as surfaces. The BC axis (dashed line) is found based on directions of blood vessels located around the BC and the orientation with respect to the pia. The column orientation is computed with respect to the C2 column. (C–E) Average dimensions of barrels and barrel columns are arranged on a grid in the layout of the barrel field. The arrows indicate the direction of the average gradient of the parameters.

However, the automated 3D reconstruction of 12 complete barrel fields, now allowed comparing the parameters of individual barrel column across the vibrissal field in a quantitative manner (**Table 1**). Barrel columns up to arc number 4 were evaluated (**Figure 4A–B**). Barrels in higher arcs were not clearly visible in all animals. We found that the five evaluated barrel column parameters varied substantially across the vibrissal cortex of individual animals (**Figure 4B–E**).

**Table 1:** Average values and variability between animals of all anatomical parameters for individual barrels and barrel columns (mean ± SD, n = 12).

Barrel	Barrel height (µm)	Barrel top (µm)	Barrel bottom (µm)	Barrel area (×10^4^ µm^2^)	Barrel volume (mm^3^)	Column height (µm)	Column diameter (µm)	Column volume (mm^3^)	Column orientation (°)
A1	322±34	455±32	777±50	8.55±1.60	0.028±0.007	1651±126	330±31	0.14±0.03	8.3±4.1
A2	337±45	467±52	805±55	8.30±1.46	0.028±0.006	1759±113	325±29	0.15±0.02	12.9±4.8
A3	315±29	485±35	800±34	6.48±1.07	0.020±0.004	1825±98	287±24	0.12±0.02	15.5±4.4
A4	316±39	489±38	805±37	6.50±1.50	0.021±0.006	1916±94	288±33	0.12±0.03	16.9±4.5
Alpha	308±39	479±53	788±48	8.79±2.50	0.028±0.010	1600±118	335±48	0.14±0.04	5.5±3.4
B1	346±34	481±48	827±56	8.74±1.59	0.030±0.006	1736±92	334±30	0.15±0.03	4.4±3.5
B2	344±27	490±45	834±47	9.01±1.48	0.031±0.005	1815±93	339±28	0.16±0.03	7.2±4.3
B3	354±29	490±53	844±57	7.66±1.07	0.027±0.004	1899±98	312±22	0.15±0.02	10.3±5.1
B4	352±21	501±52	853±52	7.89±1.15	0.028±0.005	1961±93	317±23	0.15±0.02	14.7±4.3
Beta	338±43	472±45	810±47	10.18±1.66	0.035±0.008	1623±103	360±29	0.16±0.03	6.7±2.7
C1	358±24	478±58	836±65	10.08±1.73	0.036±0.007	1800±110	358±31	0.18±0.03	6.1±4.6
C2	360±24	496±43	856±37	10.30±1.37	0.037±0.005	1892±100	362±24	0.20±0.03	0
C3	354±31	534±61	889±56	10.44±2.05	0.037±0.007	1985±100	365±36	0.21±0.04	7.3±5.1
C4	363±47	557±80	920±70	9.03±2.25	0.033±0.011	2038±94	339±42	0.18±0.05	10.3±5.2
Gamma	355±31	478±48	833±48	12.73±2.72	0.045±0.009	1713±132	403±43	0.22±0.04	11.1±5.6
D1	371±33	491±45	863±55	11.17±1.52	0.042±0.007	1865±104	377±26	0.21±0.03	7.8±4.4
D2	362±39	526±61	888±56	12.42±1.61	0.045±0.008	1957±101	398±26	0.24±0.04	5.1±4.5
D3	368±23	552±60	920±60	11.64±1.97	0.043±0.008	2046±87	385±33	0.24±0.04	3.2±2.4
D4	363±46	556±57	919±54	10.34±0.75	0.038±0.005	2081±98	363±13	0.22±0.02	5.9±3.2
Delta	354±27	506±48	860±52	14.31±3.27	0.051±0.012	1845±95	427±49	0.26±0.06	12.4±4.5
E1	343±28	546±45	888±60	14.56±2.05	0.050±0.008	1977±77	431±30	0.29±0.04	9.4±5.1
E2	355±30	557±54	912±60	15.89±1.61	0.056±0.007	2096±58	450±23	0.33±0.04	7.6±5.1
E3	364±38	549±58	912±71	15.78±2.32	0.057±0.007	2117±84	448±33	0.33±0.05	6.5±3.6
E4	344±48	580±48	924±45	12.30±2.00	0.042±0.010	2111±91	396±32	0.26±0.04	5.9±2.9

Barrel volume, column diameter and column volume are derived quantities. Colum diameter derives from a circular approximation of the barrel area.

First, BT and BB ranged from 455 µm to 580 µm and 777 µm to 924 µm distances below the pia, respectively. Both parameters varied in a codependent manner (**Figure 4C**). While the depth locations of the barrels varied across the vibrissal cortex, the barrel height was preserved (348±34 µm), suggesting that the thickness of granular L4 was constant across the vibrissal field. Consequently, the thickness of the remaining cortical layers varied. The changes in barrel depths were not random but followed a well-defined gradient (arrow in **Figure 4C**), from BC locations closer to the pia at lower row and arc numbers (minimum at A1) to BC locations deeper within the cortex at higher row and arc numbers (maximum at E4).

Second, the barrel areas displayed substantial location-specific variations across the vibrissal field, ranging from 64,800 µm^2^ to 158,900 µm^2^. The ∼2.5-fold difference in barrel area again followed a well-defined gradient (**Figure 4D** **, left panel**). However, barrel areas were smaller at lower row and higher arc numbers (minimum at A4) and increased towards higher row and lower arc numbers (maximum at E2). Because the barrel height was preserved across the vibrissal cortex, barrel volumes followed the same gradient as the barrel areas, ranging from 0.02 mm^3^ to 0.06 mm^3^ (**Figure 4D** **, right panel**).

Third, the column heights (i.e., pia-WM distance) displayed a gradient similar to the ones obtained for BT and BB (**Figure 4E** **, left panel**). The differences in average column heights were however four times more pronounced (ranging from 1,600 µm in the α-column to 2,117 µm in the E4-column) than the average differences in barrel depth. Consequently, the fraction of supragranular-to-granular-to-infragranular (s-g-i) layers was column-specific. For example, the average A1-column was 1,651 µm high. Average BT and BB in the A1-column were located at 455 µm and 777 µm, respectively. The thickness of the supragranular, granular and infragranular layers in A1 was thus 455 µm, 322 µm and 873 µm, respectively (s-g-i: 27%-20%-53%). In contrast, the average height of the E4-column was 2,111 µm. Average BT and BB were located at 580 µm and 924 µm, respectively. The thickness of the supragranular, granular and infragranular layers in E4 was thus 580 µm, 344 µm and 1187 µm, respectively (s-g-i: 28%-16%-56%). Hence, defining granular L4 by the vertical extent of the barrels [24] yielded that supra- and infragranular layers in columns with lower row and arc numbers were relatively thinner compared to L4 and relatively thicker in columns with higher row and arc numbers.

Fourth, the volumes of the barrel columns displayed a location-specific gradient, different from the ones observed for barrel areas or column heights (**Figure 4E** **, right panel**). The gradients of the barrel areas and column heights compensated each other yielding approximately constant barrel column volumes within the same whisker row (A-row: 0.12–0.15 mm^3^; B-row: 0.15–0.16 mm^3^; C-row: 0.18–0.21 mm^3^; D-row: 0.21–0.24 mm^3^; E-row: 0.26–0.33 mm^3^). Specifically, the average SD in column volume within the same row was 0.017 mm^3^. In contrast, the average SD in column volume between rows was four times larger, i.e., 0.067 mm^3^.

**Figure 5:**
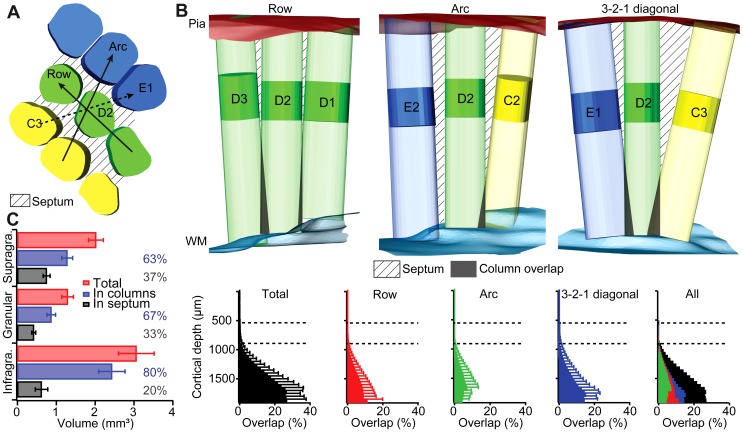
Cortical curvature leads to anisotropic overlap of barrel columns. (A) The granular layer is subdivided into barrels and the septum between barrels. The vectors describe the direction along the row (e.g., D1-D2-D3), arc (e.g., C2-D2-E2) and the 3-2-1 direction (e.g., C3-D2-E1). (B) Top: overlap of neighboring barrel columns in one reconstructed barrel cortex in different directions, based on a cylindrical extrapolation of the column. The magnitude of the overlap is influenced by the distance between neighboring columns and the magnitude of the curvature in different directions. Bottom: average values across all columns and all reconstructions. Error bars are 1 standard deviation. (C) Measurement of the average volume inside barrel columns and septa in all reconstructed barrel cortices. Error bars are ±1 standard deviation.

Finally, the orientation (i.e., vertical axis) of the barrel columns with respect to each other was not parallel, but tilted, following the curvature of the pia. We defined the vertical axis of the C2-column as the Null direction and determined the tilt of the remaining columns with respect to this axis (**Figure 4B** **, bottom panel**). All barrel columns were tilted with respect to C2. The gradient describing the column orientations across the vibrissal cortex followed a symmetric relationship along an axis approximately parallel to the medial axis of the brain (i.e., α→B1→C2→D3→E4). The tilt along this ‘1-2-3’ axis was substantially smaller (3–6°) than the tilt along the perpendicular ‘3-2-1’ axis (i.e., A4→B3→C2→D1→δ) (**Figure 5A**), which was between 8° and 16°. For example, the A4-column displayed a maximal average tilt of 17°. In contrast, the average tilt of the E4-column within the same arc was only 6°. This relationship reflects the fact that the rodent brain is elongated, resulting in a smaller curvature of the cortex along the medial axis than along an axis perpendicular to it.

The curvature of the cortex and the resulting tilts of the BC axes yielded barrel columns that started to overlap in deep cortical layers (**Figure 5B** **, top panels**), using the cylindrical extrapolation of the barrel towards the pia and WM. We quantified this overlap as a function of cortical depth for neighboring columns within the same whisker row, arc or along the 3-2-1 axis, respectively. The overlap was measured as the ratio of volume shared by neighboring columns to the total volume of a central column in 50 µm bins along the BC axis (**Figure 5B** **, bottom panels**). Barrel columns began to overlap right below the granular layer. The overlap increased monotonically with increasing cortical depths, reaching a maximum of ∼25% at the WM. The magnitude of the overlap was different along the three investigated axes. The overlap within the same whisker row reached on average a maximum of ∼10% at the WM and ∼5% within the same whisker arc. The largest cortical curvature along the 3-2-1 axis resulted in the largest overlap along this direction, reaching on average a maximum of about 15% at the WM. In consequence, the overlap between neighboring columns resulted on average in 7% smaller column volumes in infragranular layers, when compared to the cylindrical extrapolation of the barrels towards the WM.

The increasing overlap with cortical depth can alternatively be described by a depth-dependent change in volume that separates cortical barrel columns. The volume separating the barrels in L4 is commonly referred to as the septum. We adopted this terminology for the entire volume of the vibrissal cortex that was not covered by any cortical barrel column. We subdivided the entire volumes of the 12 reconstructed vibrissal cortices into voxels of size 10×10×10 µm^3^ and assigned each voxel either to a barrel column or the septum.

The resultant volume of the entire vibrissal cortex (α-δ, A1-E4) was 6.53±0.75 mm^3^, with 4.58±0.54 mm^3^ (∼70%) belonging to barrel columns and consequently 1.95±0.28 mm^3^ (∼30%) belonging to the septum. The total volume of the supragranular, granular and infragranular layers was 2.02±0.19, 1.29±0.15 and 3.06±0.46 mm^3^, respectively (**Figure 5C**). Further, the relative fraction of the septum from the total volume increased from the granular layer (33%, 0.42±0.05 mm^3^) towards the supragranular layers (37%, 0.75±0.09 mm^3^) and decreased towards the infragranular layers (20%, 0.62±0.16 mm^3^).

### Anatomical landmarks are preserved across animals

**Figure 6:**
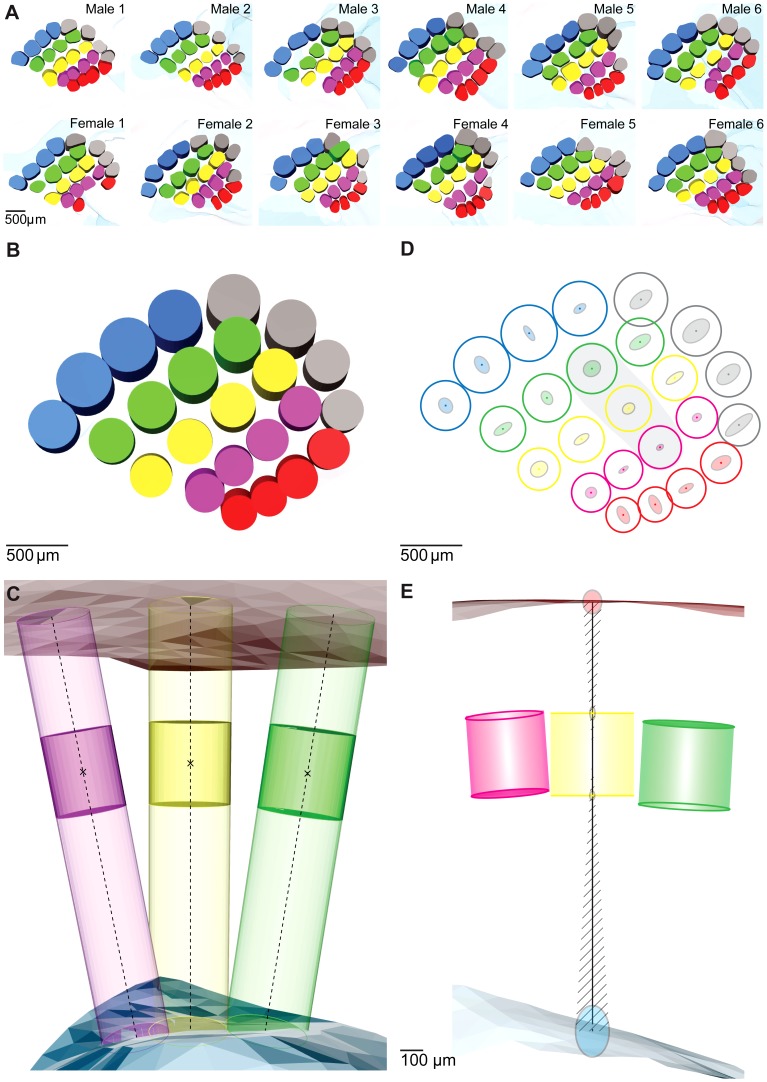
Creation of an average 3D model of the barrel cortex. (A) All barrel fields reconstructed automatically in this study. In three animals it was not possible to reconstruct all barrels, because individual barrels were not completely distinguishable from background (female 1: A2,A3; female 2: A4; female 3: γ). (B) Standardized barrels, pia and WM shown from a tangential view. (C) Three standardized barrels and barrel columns (B3, C2, D1), pia and WM shown from a (semi-coronal) side view. (D) Variability of the registered BT points measured along rows/arcs. Barrels in shaded region are shown in the side view in (E). (E) Vertical axis of the error ellipses shows the variability of the registered BT, BB, pia and WM along the barrel column axis. Dashed region indicates horizontal variability induced by variability of the column axis. Because the angular variability has a fixed value for each barrel, the induced horizontal variability increases with distance from the barrel center. This is illustrated by the horizontal axis of the error ellipses. This error is smaller than the variability along rows and arcs between animals (D), and thus negligible at the BT and BB.

We investigated whether the anatomical variability of the respective five parameters describing the dimensions of each barrel column was sufficiently small across animals (**Figure 6A**) to allow for registration of anatomical data to a standardized 3D model of the entire vibrissal cortex.

As a first qualitative assessment, we calculated the mean and SD of each parameter for each individual column **(** **Table 1** **)**. The resultant average variability of the five parameters across animals was as follows: (i) the barrel area deviated on average by 16,600 µm^2^ (SD in percent of the mean: 17%), (ii) the BT deviated by 51 µm (10%), (iii) the BB deviated by 53 µm (6%), (iv) the column orientation with respect to the C2 column deviated by 4.1° and (v) the barrel column height deviated by 98 µm (5%). Further, we checked for differences between male (n = 6) and female (n = 6) animals. Male rats had slightly, but consistently thinner vibrissal cortices (1860±185 vs.1919±171, p<0.01, 2-way ANOVA). However, none of the other barrel or barrel column parameters were significantly different (p>0.1, 2-way ANOVA). In addition, we correlated the column parameters, as well as the volume of the vibrissal cortex, with the weight of the respective animal, but found no significant relationships (p>0.05, non-directional t-test). We thus pooled all reconstructed cortices for the subsequent analyses.

To obtain a more quantitative measure of the anatomical variability of the vibrissal cortex, we registered 12 reconstructed vibrissal cortices into a common coordinate system and created an average 3D cortex model by using only rigid transformations (i.e., translations and rotations) (**Figure 6B–C**). The variability in barrel location across animals can then be determined quantitatively by computing the covariance of the 12 corresponding BT and BB coordinates with respect to the standard cortex model. Specifically, by diagonalizing the covariance matrix and computing the square root of the eigenvalues, deviations in barrel locations can be investigated along the whisker row, arc (**Figure 6D**) and BC axis (**Figure 6E**), respectively.

The average variability across BT locations in the tangential plane, as measured by the respective square root of the eigenvalues, was 67 µm along whisker rows and 49 µm along whisker arcs. The variability of the BB (row: 64 µm; arc: 48 µm) locations followed the variability of the BT (along rows: r = 0.96, along arcs: r = 0.92). In general, the variability of barrel locations in the tangential plane was much smaller than the average extent of the barrels (∼355 µm diameter) (**Figure 6D**).

The variability in barrel location along the vertical column axis is exemplarily illustrated for the C2 barrel (**Figure 6E**). There, the average variability of BT and BB locations were 28 µm and 19 µm, respectively. This is illustrated by the vertical extent of the respective error ellipses. For the remaining barrels, similarly small variability values were obtained, being on average 35 µm. Finally, combination of the 3D variability values of the BT and BB locations allowed determining the variability of the BC axes, which was 4.5° (**Figure 6E**). This variability in orientation results in an additional depth-dependent horizontal uncertainty (dashed region in **Figure 6E**) of the column location. However, this uncertainty is small compared to the horizontal variability of the BT and BB and is therefore neglected.

The variability between the 12 individually registered BT and BB locations from their counterparts in the standardized model, as measured by the respective square root of the eigenvalue, were similar to the respective average SDs determined across animals (e.g., BT: 51 µm vs. 35 µm). Hence, the precision of the BC axes in the standardized model was close to the variability in orientation across animals (i.e., 4.5° vs. 4.1°). Consequently, the rigid transformations and optimizations used to create the 3D standard model did not introduce any systematic biases. Taken together, the two quantitative measures indicate that the 3D geometry of the rat vibrissal cortex was preserved across animals and that the standardized model captures its average 3D layout.

So far, we considered the parameters from each barrel column individually, neglecting that positioning of the barrel columns with respect to each other may change between animals. We therefore introduced a set of three non-linear functions (2^nd^ order polynomials) to parameterize the 3D layout of the entire vibrissal cortex separately for each reconstructed cortex and the standardized model. The 15 coefficients of the three functions may be interpreted as specific geometrical properties of the vibrissal cortex; for example measuring the deviation of the barrel field from a rectangular grid (see Materials and Methods).

**Figure 7:**
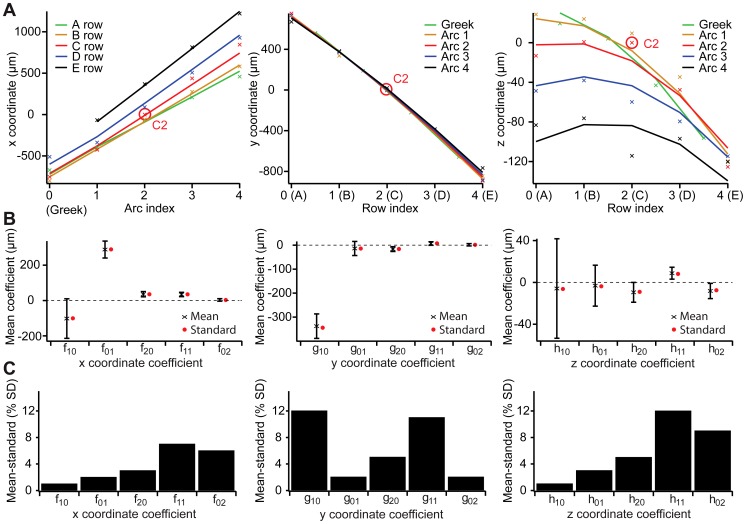
Quantitative description of the 3D anatomical layout of the barrel field. (A) Fits of 2^nd^ order polynomials in *(Row, Arc)* coordinates to the x/y/z coordinates of the BC points of the standardized barrel field. (B) Mean coefficients (black) of the fits to all reconstructed barrel fields and coefficients of the fit to the standardized barrel field (red). Error bars are ±1 standard deviation. (C) Difference between the mean coefficients and the coefficients of the standardized barrel field in units of standard deviation.

The coefficients were determined by fitting the functions to the 24 BC locations of the standardized model of the vibrissal cortex (**Figure 7A**). The fitting was further applied to each of the 12 reconstructed cortices, individually. The resulting mean and SD of each coefficient is shown in **Figure 7B**. A quantitative measure of the quality of the standard model can then be expressed as the difference between each mean coefficient and the corresponding value from the fit to the standardized barrel field (**Figure 7C**). For example: The coefficient describing the deviation of the barrel field from rectangular grid (f_10_, see Materials and Methods) was −100.77 for the fit to the standard model. Fitting the three functions to the 12 cortices individually yielded an average coefficient of −102.02±112.15. Thus, the difference between the standardized and the average coefficient was 1.25 (i.e., 102.02–100.77). Compared to the variability across animals (SD: 112.15), the difference between the two coefficients was small (i.e., ∼1% of the SD). The quality of the standard model in capturing the average 3D layout of the vibrissal cortex was hence defined in units of SD of the 15 coefficients. This measure was below 12% for all coefficients and on average around 5%.

The finding that not only the 3D dimensions of the respective barrel columns, but also the 3D layout of the entire vibrissal cortex, is preserved with approximately 5% accuracy across animals, is somewhat counter-intuitive when visually comparing individual cortices reconstructed in the present (**Figure 6A**) or previous studies (e.g. [24]). For example, the barrel shape, the size of the septum between rows (in particular between the D- and E-row) or the curvature of the arcs (in particular of the greek arc) vary between individual animals. Consequently, while the present standard model of the vibrissal cortex captures its average layout with approximately 5% accuracy (i.e., SD), the deviation of one individual barrel field from this average model may be larger.

To assess how each individual cortex reconstruction matches the average model (i.e., standard layout of the vibrissal cortex) we performed a ‘leave-one-out’ cross validation analysis. Specifically, we determined the average coefficients from only 11 cortices and then computed the root mean squared error (RMSE) between the predicted and the actual 3D BC locations of the remaining cortex. The procedure was repeated 12 times, i.e., for each reconstructed vibrissal cortex. The average RMSE was 146 µm, but varied between animals and barrel columns (**Table 2**). For example, the average RMSE of ‘Male 6’ was 187 µm, compared to 92 µm for ‘Female 1’; the average RSME for the greek arc was 187 µm, compared to 111 µm for the B-row. The latter is consistent with the analysis of BT and BB locations, as illustrated by large SD-ellipses in the greek arc and small ones at centrally located barrels (**Figure 6D**).

**Table 2:** Precision of registration for each barrel column.

Barrel	SE (µm)	SD (µm)	RMSE (µm)
A1	27	95	179
A2	24	80	170
A3	27	89	166
A4	26	86	166
Alpha	40	138	211
B1	19	65	106
B2	14	48	101
B3	15	51	108
B4	19	65	131
Beta	36	124	158
C1	27	92	106
C2	20	69	104*
C3	22	75	102
C4	30	102	167
Gamma	42	139	176
D1	30	105	153
D2	28	98	121
D3	24	83	110
D4	27	95	150
Delta	33	114	203
E1	20	71	148
E2	23	81	136
E3	26	91	160
E4	24	85	168

The average precision of the soma/dendrites/axon location within the principal column (i.e., containing the neuron's soma) is determined as the standard error of the barrel location (SE). The average precision of long-range projecting axons into columns surrounding the principal column is given by the standard deviation of the barrel location (SD). The minimal precision is derived from the leave-one-out analysis as the root mean squared error between the predicted and actual barrel location (RMSE). The RMSE of the C2 barrel (*) is computed as the average of C1 and C3, because the C2 BC is the origin of the coordinate system during parameterization.

### Precise registration of individual 3D neuron morphology

The quantifications of the variability of the vibrissal cortex and the quality of its standardized model suggest that 3D reconstructions of neuron morphologies can be registered with high precision, if the respective reference landmarks are present in each tracing. Unfortunately, the high-contrast Cytochrome-oxidase staining needed to automatically extract the barrel landmarks prevents tracing biocytin-labeled [34] dendrite and in particular axon morphologies. In turn, the low-contrast Cytochrome-oxidase staining needed to reliably trace neuron morphologies prevented us from automatically extracting the barrel landmarks. Thus, to assess how accurate 3D neuron tracings can be registered to the standard model by rigid transformations, systematic differences between manually and automatically extracted reference landmarks needed to be quantified.

**Figure 8:**
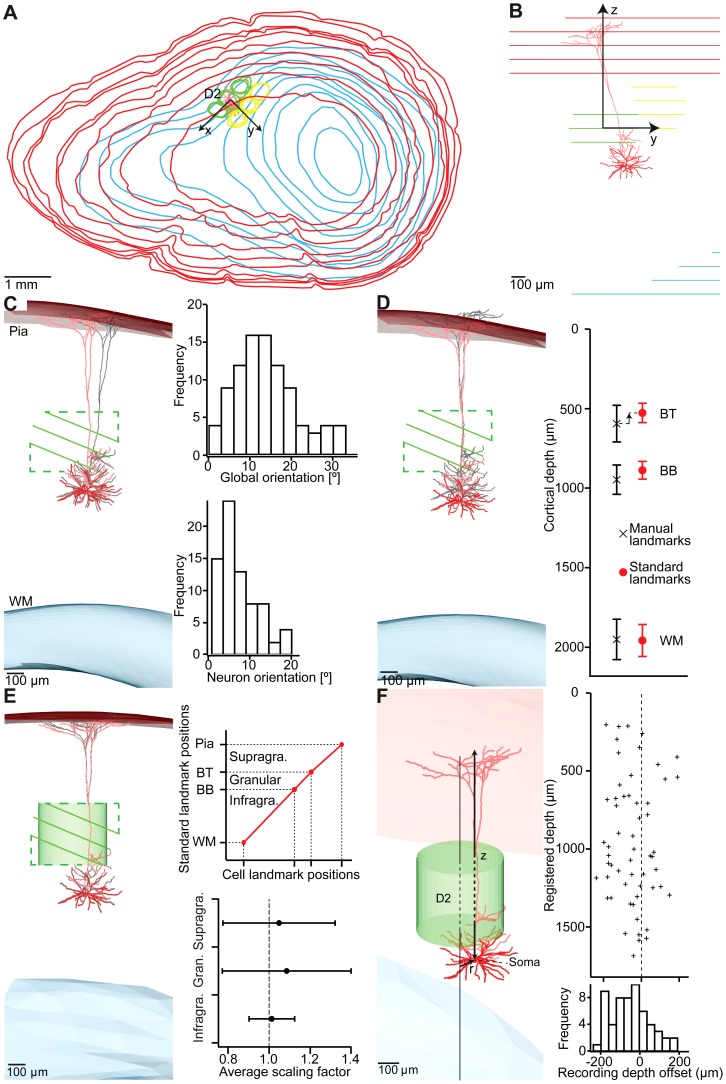
Registration of 3D neuron morphologies to the standardized barrel cortex. (A) Example of a L5 thick-tufted neuron reconstructed from 100 µm thick sections. Outlines of pia, WM and barrels are added to the reconstruction in the coordinate system given by the slicing direction. (B) Side view of (A). The slicing direction does not match the orientation of the column containing the neuron soma. (C) Reconstruction of landmarks in 3D and registration of the barrels to the standardized barrel field. It may be necessary to correct the orientation of the neuron to match the direction of the local column axis (gray – before rotation, red – after rotation). The histograms show the rotation angle used to align the barrel field outlines with the standardized barrel field (global orientation) and the angle of the subsequent rotation aligning the neuron orientation with the local column orientation. (D) The barrel outlines in the reconstruction are of lower resolution along the slicing direction and thus show a systematic offset compared to the standardized barrel landmarks. This is corrected for by translation along the local column axis. (E) The variability between different reconstructions is minimized by scaling the supragranular, granular and infragranular structures such that the landmarks of the reconstructed neuron coincide with the standardized landmarks. The average scaling factors for the individual layers are very close to 1. (F) Registration of the neuron to the standardized barrel cortex allows objective determination of anatomical parameters such as the soma location in 3D. Comparison of the registered depth of 56 neurons with the penetration depth of the pipette recorded during the experiment shows that this recording depth is on average 46 µm lower than the registered depth, but varies in a range of up to 200 µm around the registered depth.

To do so, we manually traced all visible anatomical landmarks for 94 reconstructed neuron morphologies with somata located randomly within the vibrissal cortex and at varying cortical depth between L2 and L6 (recording depth: 222–1727 µm [3]). Using this set of morphologies we developed a precise registration pipeline that automatically compensates for differences between manually and automatically extracted landmarks. The individual steps of the pipeline are exemplarily illustrated for one L5 thick-tufted pyramidal neuron [2] in **Figure 8**.

The BC of the manually reconstructed principal column (i.e., containing the neuron's soma) was aligned with the respective BC of the standard cortex. Then, the remaining BC locations were registered by using only rigid transformations (**Figure 8C** **, left panel**). This step resulted in a rotation of the principal BC axis of 14.0±7.6° (1.6–32.8°, **Figure 8C** **, top-right panel**). Because the BC axis of an unregistered tracing is defined by the cutting plane of the vibratome, the rotation of the ‘global orientation’ of the neuron compensated for systematic differences introduced by cutting the brain into sections.

The orientation of the BC axis after the first registration step was on average more variable (SD: 7.6°) than the 4.5° deviation in column orientation determined for the standard vibrissal cortex. This likely reflected the observation that the manually determined contours defining BT and BB were less precise than their automated counterparts. We thus introduced a second rotation step. The apical dendrite of pyramidal neurons in the cortex usually projects along an axis perpendicular to the pia surface and thus, parallel to the large blood vessels in its immediate surrounding [35]. The local blood vessel pattern can consequently be used to determine the vertical axis of a barrel column and hence of a reconstructed neuron. To do so, we reconstructed the blood vessels throughout the vibrissal cortex and determined local vertical axes with 50 µm precision (i.e., 50 µm spacing between neighboring vertical axes, see Materials and Methods and Text S1). Further, we determined the smallest moment of inertia of the apical dendrite and rotated the tracing until this ‘dendrite orientation’ matched the vertical axis closest to the respective soma. In cases where no clear apical dendrite was present (e.g., for L4 spiny stellate neurons [36]), the direction of the main axon leaving the soma in a straight direction towards the WM was defined as the neuron's orientation. The additional rotation of the ‘neuron orientation’ was small (0.8–20.0°, 7.3±4.5°, **Figure 8C** **, bottom-right panel**) compared to the global orientation step. In particular, the resultant variability in neuron orientation of 4.5° matched the previously determined variability in BC axis orientation across animals.

After translations and rotations, the new BT, BB, pia and WM locations were systematically compared to their counterparts in the standardized cortex model. The average vertical locations of all landmarks deviated from the standard model (**Figure 8D** **, right panel**). All parameters varied independently for different columns. For example, for the D2 column, the manual BB deviated on average 59 µm from the respective standard landmark (manual: 947 µm vs. standard: 888 µm depth below the pia surface). The BT deviated on average by 68 µm (manual: 594 µm vs. standard: 526 µm) and the depth location of the WM deviated on average by 7 µm (manual: 1950 µm vs. standard: 1957 µm) from the respective standard landmarks. Consequently, we shifted the contours of the principal column in each tracing by the respective differences between the mean values of the manual tracings and the standard cortex (**Figure 8D** **, left panel**).

Further, we measured the distance between the apical tuft endings and the reconstructed pia surface exemplarily for four neurons where the apical tufts reached the upper most part of L1 (i.e., true distance to the pia surface was zero). We found that the average distance of the apical tuft endings to the reconstructed pia surfaces was 39±5 µm. Thus, we shifted all manually traced contours by −39 µm with respect to the neuron tracing. In addition, the thickness of the first vibratome section may deviate from the assumed 100 µm thickness. We therefore compared the average distance to the pia for four neurons whose apical tufts ended within the first vibratome section and ten neurons with tufts already reaching the pia in deeper sections. We found that the reconstructed pia of the first section was on average 20 µm too high and corrected the vertical pia location accordingly.

In the final registration step, differences between the registered vertical locations of BT, BB, pia and WM of each individual neuron tracing were compared to the respective standardized landmarks (**Figure 8E** **, top-right panel**). BT, BB, pia and WM deviated independently from each other. Therefore, we chose a stepwise linear scaling to match the respective landmarks of each tracing with the standardized counterparts (**Figure 8E** **, left panel**). Three scaling factors were determined between: (i) the pia and the BT (i.e., supragranular layers), (ii) the BT and BB (i.e., granular layer) and (iii) the BB and the WM (i.e., infragranular layers). The scaling factors were on average very close to 1 (i.e., 1.05±0.27, 1.09±0.31 and 1.01±0.11 in supragranular, granular and infragranular layers, respectively).

In summary, by (i) coarse registration of BC locations, (ii) fine tuning of neuron orientation, (iii) shifting the vertical locations of BT, BB, pia and WM by their respective average differences between manually and automatically determined landmarks and (iv) stepwise linear scaling of the neuron along the BC axis, we found that the manually reconstructed vibrissal cortices could be matched to the standard cortex as precisely as the automatically reconstructed versions.

The precision of registering individual neurons to the standardized model may thus be expressed as the standard error (SE) of the average BC location as determined by the covariance matrix above, multiplied with the respective scaling values in supragranular, granular and infragranular layers, respectively. Specifically, the vertical precision of the supragranular layers can be determined as the SE of the BT locations, which was 15 µm, multiplied with the average scaling of 1.05, resulting in SE_z,supra_ = 16 µm. The vertical precisions of the granular and infragranular layers can be determined accordingly by the SE in barrel and column heights (i.e., SE_z,granular_ = 10 µm and SE_z,infra_ = 28 µm), respectively. Combined with the precisions along the row and arc (SE_row_ = 19 µm, SE_arc_ = 14 µm, see above), we obtained a 3D registration accuracy for neurons located in supragranular layers of 28 µm, in the granular layer of 26 µm and in infragranular layers of 37 µm.

Consequently, the 3D location of the soma, as well as dendrites and axons close to the principal column, can on average be determined with ∼30 µm accuracy. However, the registration was optimized to match the BC location of the principal column. The registration accuracy of neuronal branches that project out of the principal column (i.e., long-range projections into septa and surrounding columns) was hence not determined by the SE of the surrounding BC locations, but by their average SDs. The average 3D registration accuracy of neuronal (long-range) projections within surrounding columns was thus ∼89 µm.

At this stage it should be emphasized that the present registration precisions are to be considered with respect to the average dimensions of the vibrissal cortex, i.e., SE and SD of the barrel location describe the precision of registered local and long-range projections, respectively. However, since the 3D layout of an individual cortex may deviate more from the standard cortex than the average of the 12 cortices, the ‘minimal’ precision of registration may be given as the average RMSE of the BC locations from the ‘leave-on-out’ analysis, i.e., 146 µm. For a summary of the column-specific registration precisions see **Table 2**.

As a first application of the registration method, we compared the vertical locations of the somata after registration with their respective recording depths (i.e., penetration depth of the pipette, **Figure 8F**). In general, the recording depth slightly deviated from the registered depth. Some neurons were deeper within in the cortex than suggested by their recoding depths; others were closer to the pia. On average, the recording depth deviated by −46±102 µm from the registered soma depth (i.e., unregistered neurons appeared to be deeper within the cortex).

The surprisingly small difference of on average 46 µm between the registered depth of the soma and the penetration depth of the recording pipette suggest that tissue shrinkage due to perfusion, fixation and histology (see Materials and Methods), which can be up to 20% [37], is largely compensated by the present approach of generating a standard model of the vibrissal cortex. Consequently, the recording depth may be used as a predictor of a neuron's location within the present reference frame of the vibrissal cortex with approximately ±102 µm precision.

## Discussion

### Comparison with previous studies of the vibrissal cortex geometry

Various attempts to quantify the geometry of individual barrels in the rodent vibrissal cortex have been reported previously [25], [28], [29], [30]. In these studies, anatomical barrel parameters were measured in 2D using manual reconstructions on low-resolution images of single or a few consecutive brain sections, either in the tangential or thalamocortical plane. In contrast to these 2D approaches, we determined five parameters (barrel area, BT, BB, column height, BC axis) describing the geometry of almost 1,000 barrels across 104 rats in 3D.

Going beyond the scope of the previous 2D studies, we found that the five 3D column parameters varied substantially across the vibrissal cortex (e.g., the barrel area ranged from 65,000 to 160,000 µm^2^ or the cortical thickness ranged from 1,600 to 2,100 µm). Further, the differences in column dimensions were not random, but followed well-defined gradients. In contrast, the variability of the five parameters was remarkably small across different animals (i.e., the SD was usually ∼5% of the mean).

Moreover, we found that the precision of cutting the brain with exactly the same orientation into tangential sections was only around 14.0±7.6°. Hence, 2D reconstructions of the barrel geometry will likely be subject to systematic errors, because the vertical axes along which parameters, such as barrel area and height, are determined vary between preparations. Further, the curvatures of the pia and WM resulted in column orientations not parallel, but tilted with respect to each other. The tilt deviated along different axes and was most pronounced for neighboring columns along an axis perpendicular to the medial axis of the brain (i.e., 3-2-1 axis). Hence, even if the cutting angle would be identical across preparations, 2D measurements of barrel area and height will still be affected by systematic errors due to the curvatures of the cortex.

However, when evaluating the dimensions of only a single column, the tilt of the neighboring columns can be neglected and systematic errors in cutting angle may be compensated by large numbers of reconstructed barrel columns. Thus, the previously reported dimensions of the D2 column in rats, based on 2D tracings of axonal projections from the posterior medial division of the vibrissal thalamus (POm) [25], were in remarkably good agreement with the respective dimensions reported here, based on automated 3D reconstructions of Cytochrome-oxidase stained barrels (i.e., barrel area: 124,000 vs. 124,000 µm^2^ and barrel column height (i.e., pia-WM distance): 1,977 vs. 1,957 µm).

### Comparison with previous standardization approaches

Several attempts to create 3D reference frames for precise registration of single neuron morphologies have been reported for various animal models previously.

For example, reconstructing stereotypical anatomical landmarks from multiple complete brains resulted in an average 3D reference frame of the entire bee brain [18]. Using nonlinear deformations and averaging of 3D label fields, individual 3D neuron morphologies could be registered by matching the labeled landmarks to the standardized *3D Bee Brain* [18]. Similar approaches have been reported for other insect models, such as the *Drosophila brain* [16].

While the general idea of (i) determining the 3D dimensions of stereotypic anatomical landmarks, (ii) generating an average 3D model from these landmarks and (iii) registering neurons by matching anatomical landmarks to the average model are similar between the insect models and the model of vibrissal cortex presented in this study, there is one major difference: The registration to the insect brains uses non-rigid transformations (i.e., nonlinear deformations of 3D label fields), while our registration approach was based on rigid transformations (i.e., translations, rotations and stepwise linear scaling).

Typically, the 3D anatomical layout and even the number of neurons, as well as the 3D dendrite/axon projection patterns of individual neurons are stereotypic across insect brains [19], [20]. The use of label fields and nonlinear deformations may thus be justified for the reconstruction of average anatomical models, if the 3D structure of the brain of interest is sufficiently stereotypic [16].

However, the mammalian cortex is different. Neither the numbers of neurons (e.g., per cortical column [37], [38]), nor the 3D dendrite and in particular axon projection patterns [2], [3], [39] display such large degrees of stereotypy across animals. Thus, in the case of the vibrissal cortex, we argue that nonlinear deformations would certainly result in a perfect match of all anatomical landmarks, but the resultant non-rigid transformations of neuron tracings may introduce uncontrollable systematic morphological changes (e.g., in path length or innervation volume).

The variability across animals of all parameters describing the 3D layout of the vibrissal cortex was sufficiently small to create an average cortex model. Further, the set of linear transformations, introduced here, was sufficient to create a standard model, which captured the average 3D layout of the vibrissal cortex. Specifically, we showed that all parameters describing the 3D layout of the standard model were very close to the respective parameters averaged across all reconstructed cortices (i.e., SD within 5% of the mean). Thus, the precision of the standard model was basically identical to the variability between animals. Therefore, the standard model can be regarded as an optimal reference frame for the vibrissal cortex. Finally, the precision of soma/dendrite/axon locations after rigid registration to the standard cortex was on average ∼30 µm within the principal column and ∼90 µm in surrounding columns, but at least ∼140 µm (see **Table 2** for column-specific values). The registration accuracy was hence in the range of the anatomical variability of the vibrissal cortex across animals.

In conclusion, lacking a sufficiently high density of reproducible anatomical landmarks, non-rigid deformations would artificially minimize the measured, true anatomical variability of the vibrissal cortex across animals, but would not improve the accuracy of the registration. Moreover, non-rigid transformations would deform the morphology of the cortical neurons, changing their path lengths, innervation domains and even electrotonic properties [40] in an uncontrollable manner. Thus, in the case of the mammalian cortex, non-rigid transformations should be replaced by rigid ones when the true anatomical variability across animals is known and sufficiently small.

Recently, a first attempt to register neuron morphologies to the mammalian brain has been reported, using a 3D model of the hippocampus in rats [41]. There, 3D reconstructions of two hippocampi were obtained by manually tracing anatomical outlines from low-resolution images of several consecutive 16 µm thick brains sections. Registration of individual neuron morphologies was then performed by placing the somata at the recording location, determined by the coordinates of the pipette, and correction of dendritic orientation and scaling. This approach renders an important step in standardizing this large structure in the rat brain. Our results suggest, however, that the recording location *in vivo* can be systematically biased, and that large sample sizes may be required to estimate the underlying anatomical variability.

Finally, magnetic resonance imaging (MRI) has been used to generate anatomical reference frames with voxel dimensions of ∼60 µm *in vitro* [42] and ∼100 µm *in vivo* [43]. While MRI allows imaging the entire rodent brain at once, the limited spatial resolution, at present, prevents from using this imaging technique to register individual 3D neuron morphologies to the vibrissal cortex with sufficient precision to determine structural overlap between axons and dendrites.

### Conclusion

Here we presented a novel, largely automated approach to (i) reconstruct the precise 3D geometry of the vibrissal cortex in rats, (ii) generate a standardized average cortex model and (iii) register dendrite and axon morphologies obtained from *in vivo* preparations to the standard vibrissal cortex. Our results yielded five major insights:

First, the automated reconstruction of the barrel cortex geometry from high-resolution image stacks allowed extracting five parameters describing the geometry of each barrel column with higher precision than manual reconstructions. This allowed estimating the ‘true’ biological variability of column geometry within the vibrissal cortex and across animals. Second, the parameters of a respective column and the 3D layout of the entire vibrissal cortex were remarkably preserved across animals. This allowed generating a standard model that captured the average layout of the vibrissal cortex. Third, the accuracy of the standard model resembled the variability across animals, which rendered the maximal precision possible for registering single neuron morphologies. Fourth, the rigid registration approach allows placing soma/dendrites/axon at their true cortical position with ∼30 µm and ∼90 µm precision within the principal and surrounding columns, respectively.

Finally, the dimensions and orientations of individual barrel columns varied substantially across the vibrissal cortex, following well-defined gradients. This finding raises the question whether a cortical barrel column can be regarded as a stereotypical anatomical unit of the vibrissal cortex. In particular, two findings argue against this theory. First, the cortical column volume increases from the A- towards the E-row by ∼2.5-fold. Previous studies demonstrated that the average neuron density is rather constant across cortical columns [37], [38]. Hence, assuming an average neuron density of 80,000 neurons per cubic millimeter, the number of neurons would increase from ∼10,000 per column in the A-row to ∼25,000 per column in the E-row. Second, the curvature of the pia and WM surfaces resulted in tilted orientations of the BC-axes, converging towards the WM. Consequently, cylindrically extrapolated barrel columns started to overlap in deeper layers, sharing up to 25% of their volume with their surrounding barrel columns at the WM.

Thus, the column-specific (i) volume, (ii) number of neurons, (iii) overlap with surrounding columns and (iv) relative proportion of supragranular-to-granular-to-infragranular layers suggest that each barrel column is a unique anatomical and potentially functional unit, as was suggested previously by functional measurements in different barrel columns in freely behaving mice [44]. Averaging of barrel column dimensions across different whisker rows and arcs may therefore be unjustified. In contrast, the geometry of the entire vibrissal cortex is remarkably stereotypic across animals. This suggests that the vibrissal cortex itself may be regarded as an anatomical and functional unit.

## Materials and Methods

### Sample preparation

All experiments were carried out in accordance with the animal welfare guidelines of the Max Planck Society and VU University Amsterdam, the Netherlands.

Neurons were filled with biocytin in urethane-anaesthetized or fentanyl-sedated Wistar rats either extracellularly by using juxtasomal recording and electroporation [45] or via whole-cell recording [46]. Spiking profiles [47], [48] and morphology of these neurons have been published previously [3]. The recorded neurons were targeted with standard patch electrodes (5 MΩ) that were positioned at ∼35° with respect to midline. Vibratome sections were cut approximately tangential to the barrel field by positioning the brains at an angle of ∼45° with respect to midline. Neurons were revealed with the chromogen 3,3′-diaminobenzidine tetrahydrochloride (DAB) [34]. Dendrite and axon morphologies were obtained between postnatal days 25–35. Automated barrel field reconstructions were obtained at postnatal day 28. Animal weights ranged from 68 g to 93 g (mean 77±8 g). No obvious differences in morphologies and cortex dimensions were observed at different ages and weights.

Cytochrome-oxidase staining was performed on 50 or 100 µm thick sections using phosphate-buffered saline (0.05 M) containing 0.2 mg/ml Cytochrome C (Sigma), 0.2 mg/ml catalase (Sigma) and 0.5 µg/ml DAB. To perform manual tracings of barrel outlines in Cytochrome C positive sections, Cytochrome-oxidase staining was performed for 45–60 minutes at 37° C. For automated detection of barrels, Cytochrome-oxidase staining was performed overnight at 37°C.

### Data acquisition

Neuron tracings were performed on 50 or 100 µm thick vibratome sections, cut approximately tangential to the D2 barrel column. Ranging from the pia surface to the white matter, 40 or 24 sections were reconstructed per neuron. DAB-stained dendrites were detected manually using Neurolucida software (MicroBrightfield, Williston, VT, USA). Axons were detected and traced in each brain section using a previously described automated method [49], [50]. Manual post-processing of individual sections [51], as well as automated alignment of reconstructed branches across sections [52], were performed using a custom-designed 3D editing environment based on ZIBamira visualization software v2010.06 (Zuse Institute Berlin). Pia and barrel outlines were manually traced in each section at low resolution (Olympus 4× UPLAN S APO; 0.16 NA) and added to the tracings in Neurolucida software (MicroBrightfield, Williston, VT, USA).

A standard transmitted light brightfield microscope (Olympus BX-52, Olympus, Japan) equipped with a motorized x-y-z stage (Märzhäuser, Wetzlar, Germany) was used for automated mosaic/optical-sectioning image acquisition, using Surveyor Software (Objective Imaging Ltd, Cambridge, UK). A 435±70 nm band-pass illumination filter, was attached to the diaphragm of the lighthouse to provide high contrast of the barrels. A 4× air objective (Olympus 4× UPLFLN; 0.3 NA) with a pixel size of 2.33 µm was used for reconstruction of pia, WM and blood vessels. A 40× oil immersion objective (Olympus 40× UPLFLN; 1.3 NA) with a pixel size of 0.23 µm and optical sectioning of 1 µm spacing was used for reconstructing the barrel field (**Figure 1C**, usually in 11–13 sections). Individual image planes were down-sampled to a pixel size of 1.85 µm.

### Image processing

All processing was carried out on workstations with Intel Xeon processors (8 cores/12GB RAM) or compute-servers with Intel Xeon processors (24 cores/256GB RAM). Segmentation and reconstruction of blood vessel, pia, WM and barrel outlines was performed automatically using custom written C++ routines, in part based on ITK [53], VTK [54], OpenMP and GSL [55] libraries. All image processing algorithms, filters and parameters were determined by systematic testing. The individual steps of the image processing and 3D reconstruction pipelines are described in detail in the Supplemental Materials (**Figure S1, S2, S3, Text S1**).

Briefly, blood vessels are automatically extracted from low-resolution images and median projections of the high-resolution image stacks. Outlines of the pia and WM are automatically extracted from low-resolution images in each brain section using thresholding and region growing methods (**Figure S1**).

Barrel outlines are automatically detected in each optical section of the high resolution image stacks during three processing steps (**Figure 2**). First, a set of gray value-based filters enhances the contrast between the barrels and the septum, by reducing the noise introduced by structures at small spatial frequencies (e.g., blood vessels or unstained neuron somata, **Figure 2D–F** **, Figure S2**). Second, seed points are manually assigned for each barrel in one brain section where all barrels are visible (**Figure 2A**). Based on these seed locations, Voronoi Regions (VR) are calculated as a first order approximation of each barrel (**Figure 2B**). Within each VR, a set of landmark-based segmentation filters (**Figure S2**) extracts the barrel circumference for each optical section (**Figure 2B–I**). Third, the quality of each barrel contour is evaluated by set a model-based correction filters (**Figure 3A–C** **, Figure S2**). This step guarantees that barrel diameters decrease towards the respective BT and BB points and again increase outside the barrels. Consequently, the vertical extent of each barrel can be objectively determined as local minima in barrel diameter (**Figure 3D–E**).

All automatically extracted contours (i.e., vessel, pia, WM, barrel) are converted into closed graphs. By manually or automatically matching the extracted blood vessel patterns [50], [52], the contours from all low- and high-resolution images from the same animal are aligned and merged into a single file. Application of 2D distance transforms to the individual sections allows transforming the pia and WM contours into 3D isosurfaces, respectively (**Figure 4** **, Figure S3**).

Using the pia surfaces, the orientation of the blood vessels is determined. Vessels that are not perpendicular to the pia (i.e., angle between the vessels and the normal vector of the pia triangle at the intersection point is larger than 10°) are deleted. The remaining vessels are used to constrain the vertical BC axes. For each BC, a set of candidate axes is determined for each triangle of the pia surface within a 2 mm radius. The quality of each axis is scored. The shorter the axis and the more perpendicular to the pia surface, the higher the score. Finally, from all candidate axes that are parallel to the average vessel orientation within the respective barrel, the one with the highest score is automatically chosen as the BC axis.

Finally, the reconstructed barrel contours are projected to the respective BC axis, defining the BT and BB points. Calculating the average barrel circumference and extrapolating it towards the pia and WM, completes the reconstruction pipeline and allows extracting the five parameters per column needed to quantitatively describe the 3D geometry of the rat vibrissal cortex (BT, BB, barrel area, BC axis and pia-WM distance).

### Registration and standardization of the vibrissal cortex

The first step in generating a standard barrel cortex is registration of all reconstructions to a common coordinate system. Only translations and rotations are used for registration. Corresponding BT and BB points from all reconstructions are used to align different reconstructions. Further, the BC axis passes through these points. Aligning all corresponding BT and BB points therefore implicitly aligns the BC axes from different reconstructions. The transformations for each reconstruction are computed by minimizing the sum of squared differences *S* between BT and BB points of corresponding barrels for all reconstructions: 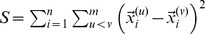. Here, *i = 1,2,…,n* enumerates the corresponding BT and BB points and *u,v = 1,2,…,m* refer to different reconstructions to be matched at each corresponding point *i*. This is equivalent to minimizing the sum of squared differences between the BT and BB points of all reconstructions and the centroids of the corresponding points of all reconstructions [56]. An analytic solution to this problem exists. However, because the centroid itself depends on the desired transformations for each reconstruction, an iterative algorithm is used to find an approximate solution [56].

Briefly, one barrel reconstruction is arbitrarily selected as a reference. For all other barrel reconstructions, the optimal translation and rotation with respect to the reference reconstruction are computed separately. The centroids of all corresponding points are computed and used as reference during the next iteration. For every iteration step the optimal translation is given by aligning the overall centroid of the reference with the overall centroid of the reconstruction to be matched. The optimal rotation is then computed from the singular value decomposition of the product of the two point position matrices set up from the positions of all BT and BB points of the reference and the reconstruction to be matched. No scaling was allowed. Only one iteration step was necessary, because the change in BT and BB positions was less than 1 µm after the first iteration.

The position of the BT and BB points of each barrel in the standardized cortex model is set to the average centroid of the respective BT and BB after registration, resulting in average BC axes. In addition, a vector field representing the local orientation is created with a resolution of 50×50×50 µm^3^ voxels. The vector at each voxel is computed by linear interpolation of the orientation of the three nearest BC axes.

The standard barrel contour is created as a circle with cross-sectional area equal to the average cross-sectional area of the respective barrels (**Table 1**). The standard barrel columns are created by extrapolation of the barrel contours along the standard barrel axes by the average distances of the barrels to pia and WM (**Table 1**). This approach is justified, because only length- and angle-preserving transformations are applied to the individual reconstructions before computing the average barrel field. The resultant top and bottom points of all standard barrel columns are triangulated, yielding the standard pia and standard WM, respectively (**Figure 5B,D**).

When registering neuron morphologies to the standard cortex, the BC of the principal column (i.e., containing the neuron's soma) is aligned to the respective BC in the standard model of the vibrissal cortex. The remaining registration steps (i.e., minimizing the squared differences of all BC locations to obtain the optimal rotation angle) are as for generating the standard cortex. This method was chosen to guarantee the highest possible registration accuracy of soma/dendrites/axon within the principal column, at the cost of achieving less precision in surrounding columns (see Results).

### Quantification of the 3D layout of the vibrissal cortex

The somatotopic layout of the barrel field in rows and arcs can be described as a map from the 2D discrete *(Row, Arc)* space to a 3D one, using the centroid location of each barrel. To do so, we chose a coordinate system as shown in **Figure 1B**. The origin is located at the centroid of the C2 barrel. The z-axis points vertically along the C2 BC axis, the x-axis points towards the centroid of the C3 barrel, approximately along the row. Thus, the y-axis points approximately along the arc. Using this description, the layout of the 12 registered and the standardized barrel field is described by *24×3 = 72* parameters. The 2D–3D mapping of the barrel cortex layout is then described by three functions *f*, *g*, *h* of row and arc index: 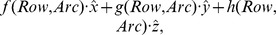 where 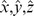 are unit vectors along the axes of the coordinate system. The three functions are modeled as polynomials of 2^nd^ order, i.e., they are of the form: 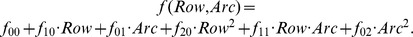


Linear functions proved insufficient to describe the non-linear spacing between rows or the curvature of the barrel cortex. Higher-order polynomials showed no obvious improvement in the description of the 3D layout of the vibrissal cortex. The 15 coefficients of the three functions describe different features of the barrel field layout: The linear coefficients *f_01_* and *g_10_* describe regular spacing of arcs and rows along the x- and y-axis, respectively. The linear coefficients *f_10_* and *g_01_* describe the relative shift between barrels in neighboring arcs or rows and can be used to measure the deviation of the barrel field layout from a rectangular grid. The second-order coefficients of *f* and *g* describe nonlinear effects such as curved rows and arcs or septa of different sizes between rows. The coefficients *h_ij_* describe the cortical depth of the BC points. The constant coefficients can be neglected.

For a numerical description of the 15 coefficients, the *Row* and *Arc* coordinates of each barrel are mapped on integer numbers, such as *A row→0*, *B row→1*, *greek arc→0*, *arc 1→1*, etc. The barrels of the greek arc are mapped on half-integer *Row* coordinates. The coefficients are determined by fitting the functions to the barrel centroids (**Figure 7**).

## Supporting Information

Figure S1 **Automated segmentation of anatomical landmark contours from 4× images (see also** **Figure 1E** **).** Blood vessels – orange; pia – red; WM – blue. Abbreviations: T – threshold; px – pixel; N – pixel neighborhood; BG – background; μ – mean; σ – standard deviation; I′ – pixel intensity after mapping; I – pixel intensity before mapping; n – number of pixels; N_15_/μ_15_/σ_15_ – number, mean and standard deviation of all pixels in a 15×15 neighborhood around the central pixel.
(TIF)

Figure S2 **Automated segmentation of barrel contours from 40× image (see also** **Figures 2** **–** **3** **).** Abbreviations: TH – top hat; VR – Voronoi region; LM – landmark; CC – connected component; r_45/85_ – quality of circles with radius of 45/85 pixels.
(TIF)

Figure S3 **3D reconstruction of anatomical landmarks from 2D contours (see also** **Figures 4A–B** **).** Abbreviations: S01 – vibratome section 01; d/d_(x,y)_ – value of distance transform (at position x,y); α(…, …) – angle between two vectors; v – vessel orientation vector; n_pia_ – normal of pia surface triangle; r – distance of vertex to vertical axis/BC axis; d_BC_ – distance from BC to pia surface triangle.
(TIF)

Text S1 **Detailed description of the image processing and 3D reconstruction pipeline.** The pipeline shown in Supplemental Figures S1, S2 and S3 is described in more detail to facilitate re-implementation.
(DOCX)
