# Supplementary figures and images for: 3D Reconstruction and Standardization of the Rat Vibrissal Cortex for Precise Registration of Single Neuron Morphology
Source: PLoS Comput Biol. 2012 Dec 20;8(12):e1002837. doi: 10.1371/journal.pcbi.1002837 (PMC3527218; doi:10.1371/journal.pcbi.1002837)

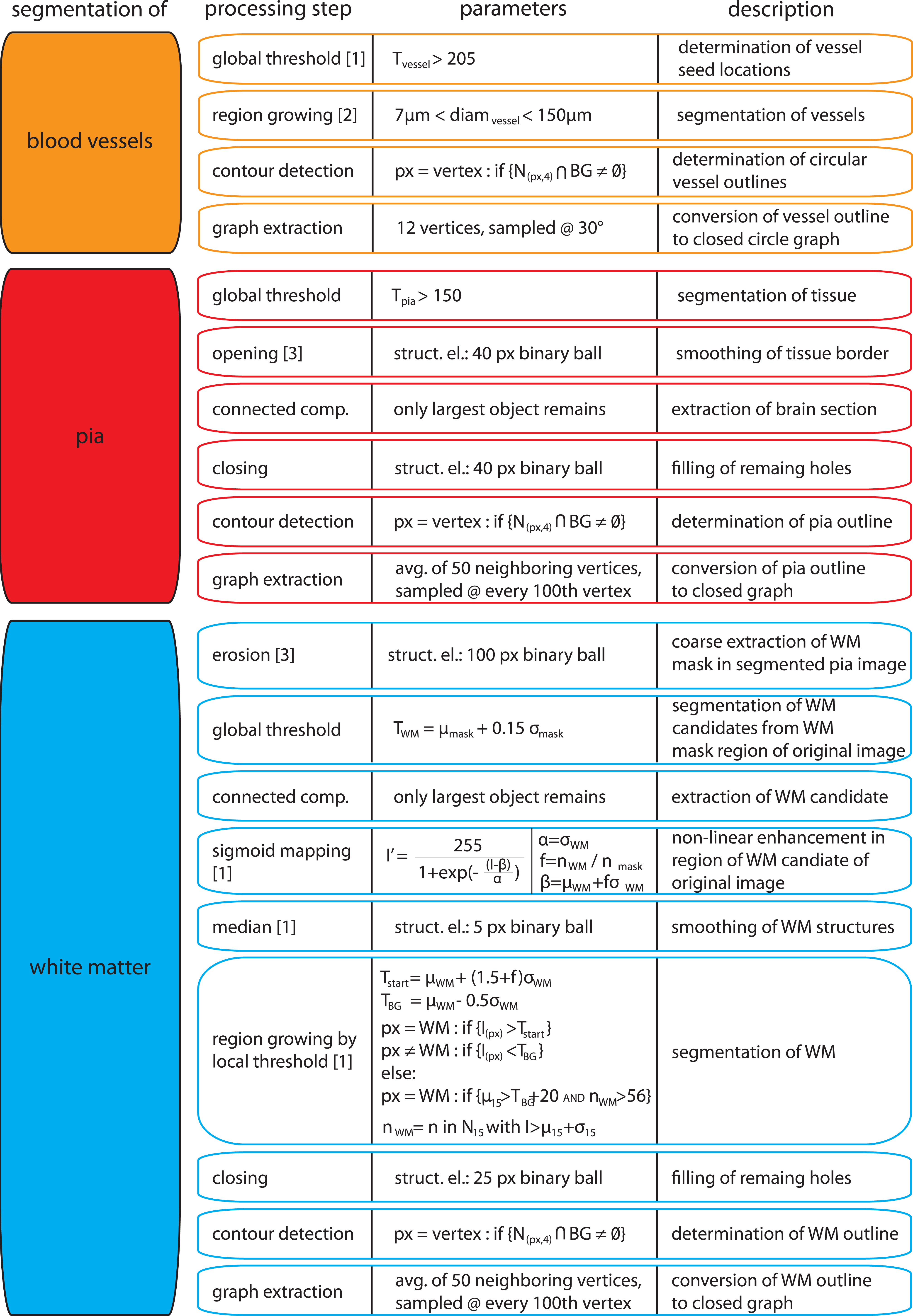

Supplement: Figure S1 — Automated segmentation of anatomical landmark contours from 4× images (see also Figure 1E ). Blood vessels – orange; pia – red; WM – blue. Abbreviations: T – threshold; px – pixel; N – pixel neighborhood; BG – background; μ – mean; σ – standard deviation; I′ – pixel intensity after mapping; I – pixel intensity before mapping; n – number of pixels; N15/μ15/σ15 – number, mean and standard deviation of all pixels in a 15×15 neighborhood around the central pixel. (TIF) [file pcbi.1002837.s001.tif]

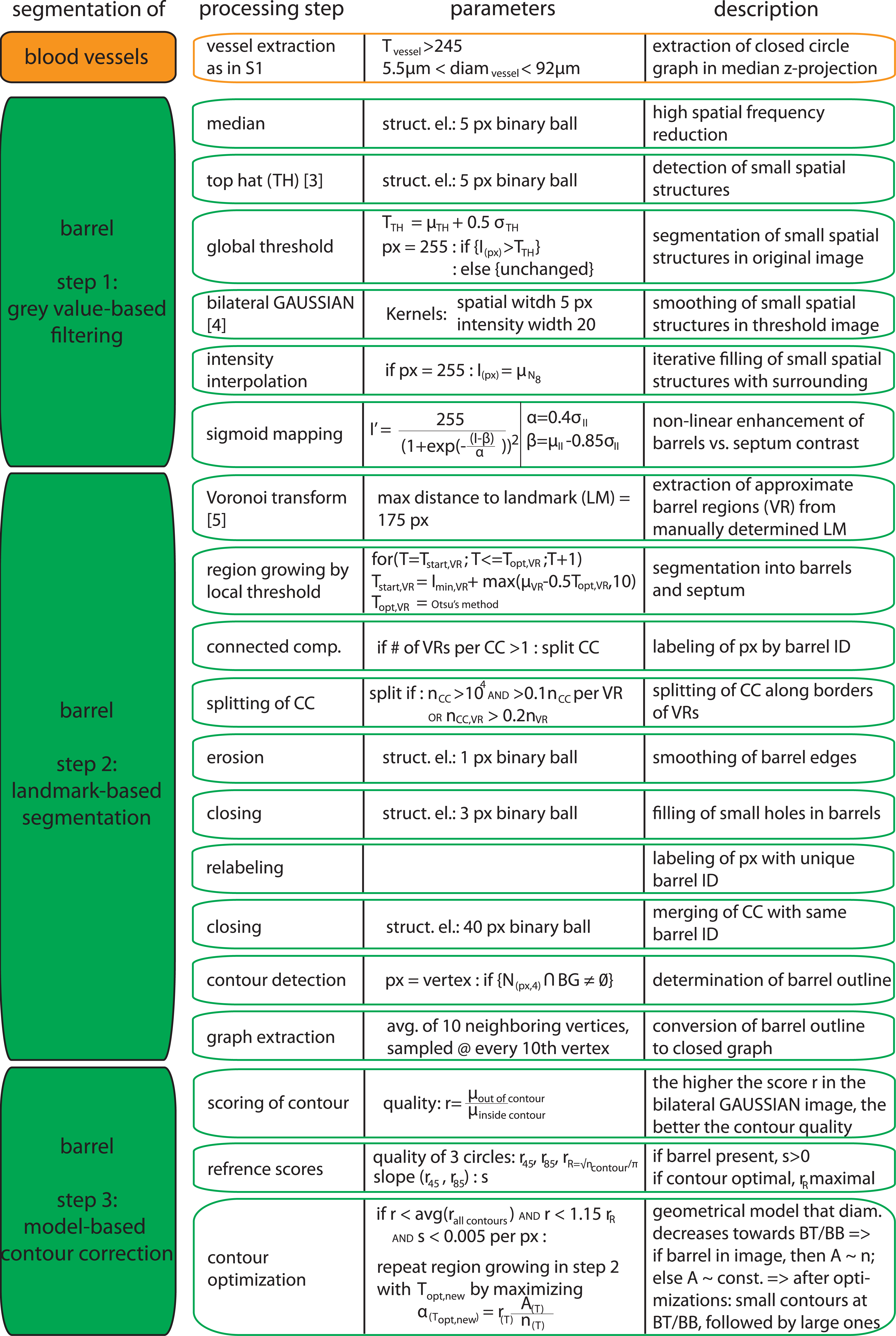

Supplement: Figure S2 — Automated segmentation of barrel contours from 40× image (see also Figures 2 – 3 ). Abbreviations: TH – top hat; VR – Voronoi region; LM – landmark; CC – connected component; r45/85 – quality of circles with radius of 45/85 pixels. (TIF) [file pcbi.1002837.s002.tif]

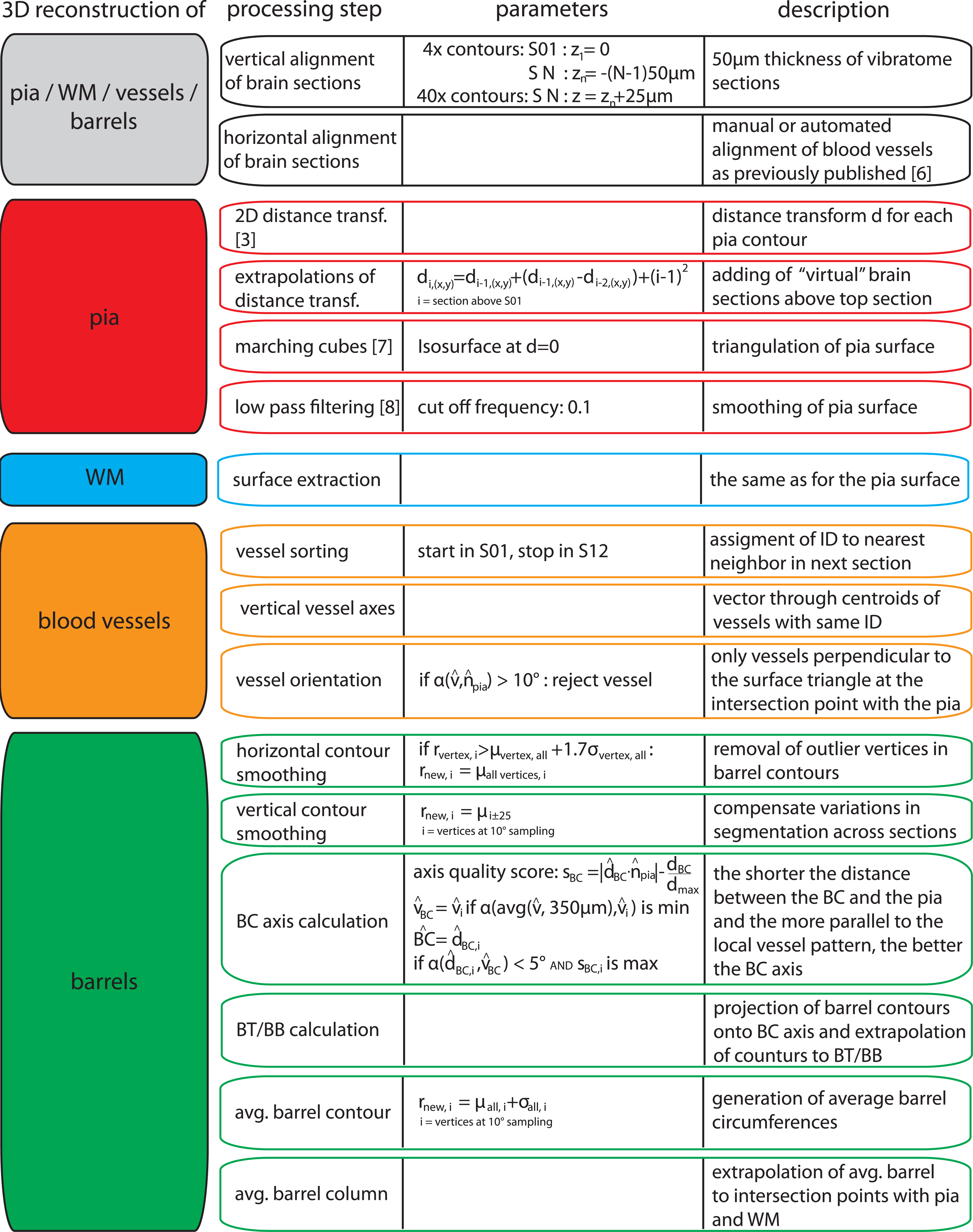

Supplement: Figure S3 — 3D reconstruction of anatomical landmarks from 2D contours (see also Figures 4A–B ). Abbreviations: S01 – vibratome section 01; d/d(x,y) – value of distance transform (at position x,y); α(…, …) – angle between two vectors; v – vessel orientation vector; npia – normal of pia surface triangle; r – distance of vertex to vertical axis/BC axis; dBC – distance from BC to pia surface triangle. (TIF) [file pcbi.1002837.s003.tif]
