# Supplementary material for: 3D Reconstruction and Standardization of the Rat Vibrissal Cortex for Precise Registration of Single Neuron Morphology
Source: PLoS Comput Biol. 2012 Dec 20;8(12):e1002837. doi: 10.1371/journal.pcbi.1002837 (PMC3527218; doi:10.1371/journal.pcbi.1002837)
Supplement: Text S1 — Detailed description of the image processing and 3D reconstruction pipeline. The pipeline shown in Supplemental Figures S1, S2 and S3 is described in more detail to facilitate re-implementation. (DOCX) [file pcbi.1002837.s004.docx]

**Text S1**

**Detailed description of the image processing and 3D reconstruction pipeline**

In the following, the pipeline shown in Supplemental Figures S1, S2 and S3 is described in more detail to facilitate re-implementation. All software is custom-written in C++ using the ITK (v3.20, http://www.itk.org/), VTK (v5.6.0, <http://www.vtk.org/>) and OpenMP (http://openmp.org/wp/) libraries. The segmentation of contours from the images/image stacks (**Figure S1** and **Figure S2**) uses the ITK and OpenMP libraries, while the 3D reconstruction of anatomical structures **(Figure S3)** uses the ITK and VTK libraries. (N.B.: The numerical methods supplied by the GSL are only used for computation of the optimal rotation matrices during registration of different barrel field reconstructions and neuron morphology reconstructions and are not required here)

**Segmentation of pia, WM and blood vessels *(Supplemental Figure S1)***

Technical note:

The ITK libraries are used for handling of image data structures, image filters and pixel- and pixel-neighborhood iterators. The OpenMP library is used for parallelization of non-sequential processing steps to decrease computation time and not strictly required.

Detailed description:

The pia forms the circumference of the tangential sections. Therefore, it is necessary to segment the tissue outline of each section. This is achieved using a pipeline of several image processing operations. All parameters used in the following steps were obtained through systematic testing.

*Global threshold*: The gray value image is turned into a binary image by applying a fixed global threshold T = 150 [1]. Pixels with gray values above this threshold are set to background, while pixels below this threshold are set to foreground.

*Opening*: A morphological opening [3] is applied to the binary image to smooth the threshold contour and remove small artifacts. The structuring element is a binary ball with a radius of 40 pixels.

*Connected component filter*: All connected components [1] in the binary image are detected and all except for the largest, which corresponds to tissue, discarded. This step removes artifacts that may remain after the opening operation.

*Closing and hole filling*: A morphological closing filter [3] is applied to the image. This leads to further smoothing of the contour and removes artifacts by filling small holes in the tissue. The structuring element is a binary ball with a radius of 40 pixels.

*Contour detection*: The contour of the tissue, which is now represented by a single connected component, is detected by marking all pixels that have at least one background pixel in their 4-neighborhood [1]. This ensures that the contour is represented by pixels which are connected in their 8-neighborhood.

The WM has a different optical density from the surrounding tissue and therefore a different gray value distribution. For objective separation from surrounding tissue, however, a different image processing pipeline is used:

*Foreground threshold*: Based on the binary image used for detection of the pia contour, a *foreground mask* is computed: The morphological erosion [3] of this binary image with a binary ball with radius of 100 pixels yields a region of interest that covers only tissue. The mean μ_fg_ and standard deviation σ_fg_ of all gray values in this region of interest are used to compute a foreground threshold:

$$T=\mu_{fg}+0.15\sigma_{fg}.$$

Pixels in the region of interest with gray values above the threshold correspond to less dense tissue and WM. A connected component filter is applied and all objects except for the largest are discarded. This gives a *WM mask* for the region of interest corresponding to WM and surrounding bright tissue.

*Sigmoid intensity mapping*: The WM mask is used to calculate the mean μ_WM_, the standard deviation σ_WM_ and the ratio *f* of all pixels belonging to the WM mask to all pixels belonging to the foreground mask. All pixel intensities are now mapped with a sigmoid function:

$$I'=\frac{255}{1+\exp\left(-\frac{(I-\beta)}{\alpha}\right)}.$$

Here, *I* is the pixel intensity before the mapping and *I’* is the pixel intensity after the mapping. The parameters α and β describe the width and the center of the sigmoid function and are calculated as follows:

$$\alpha=\sigma_{WM},\beta=\mu_{WM}+f\sigma_{WM}.$$

Increasing the center of the sigmoid function β with an increasing number of WM mask pixels, *f*, is necessary to prevent oversegmentation in case there are a large number of bright pixels.

*Smoothing*: A median filter [1] with a radius of 5 pixels is applied to the mapped image to remove high-frequency variations in gray values in the WM.

*Region growing*: The WM is segmented from the smoothed image using an iterative region growing algorithm. A starting region is determined using an upper threshold:

$$T_{start}=\mu_{WM}+(1.5+f)\sigma_{WM}.$$

The factor *f* is again necessary to prevent oversegmentation in case there are a large number of bright pixels. From this starting region, neighboring pixels are iteratively processed and labeled as belonging to or not belonging to WM. A pixel is always considered belonging to WM if its intensity *I* is greater than an upper threshold. A pixel is automatically considered not belonging to WM if its intensity *I* is less than a lower threshold:

$$T_{bg}=\mu_{WM}-0.5\sigma_{WM}.$$

Every pixel with gray value *I* between the lower and upper threshold is considered belonging to WM if:

$$\mu_{15}>T_{bg}+20\wedge N_{fg}>56.$$

N_fg_ is the number of pixels in a 15x15 pixel neighborhood whose gray values are greater than:

$$\mu_{15}+0.3\sigma_{15},$$

where μ_15_ and σ_15_ are the mean and standard deviation of the gray values in this neighborhood.

At the border of the WM, the 15x15 neighborhood includes both bright WM and darker background pixels, thereby increasing σ_15_. Together with the lower threshold T_bg_, this requirement ensures that the region growing stops at the WM border.

*Closing*: A closing operation with a binary ball of radius 25 is performed on the segmented image to smooth the segmented image and fill small holes.

The WM contour can now be detected in the same way as the pia contour.

Cortical blood vessels are visible as bright circular or elliptical regions. They are detected using a region growing algorithm accepting bright circular structures with a diameter in the range of 7-115μm [2].

The contour pixels of pia, WM and blood vessels are now converted into vertices to create a common graph-based format of all contours for subsequent processing. The vertices are turned into a graph by linking neighboring vertices by edges. The topological structure of the contour (i.e., circular) is obtained by searching for the shortest possible path of a vertex leading back to itself using Dijkstra’s algorithm [9]. Next, the coordinates of the vertices of the pia and WM contours are smoothed by replacing the coordinates of every vertex by the average of the 50 neighboring vertices. In a final step, every 100^th^ vertex is sampled. Blood vessels are represented as circles. The resulting contours are shown in **Figure 1E**.

For 3D reconstruction of the cortex, all vertices are converted to coordinates in 3D space by multiplying the xy-coordinates of each vertex with the lateral sampling rate of the images. Assuming that tissue loss during the slicing process is negligible, the location of the contours of different sections along the slicing direction (z) is recovered by spacing them 50μm apart. The alignment of the contours of adjacent sections is achieved by matching the blood vessel pattern in these sections using rotations and translations in the x-y plane. After the pairwise alignment of all sections, all contours are assigned unique labels corresponding to the represented anatomical structure.

**Segmentation of barrels *(Supplemental Figure S2)***

Technical note:

The ITK libraries are used for handling of image data structures, image filters and pixel- and pixel-neighborhood iterators. The OpenMP library is used for parallelization of non-sequential processing steps to decrease computation time and not strictly required.

For automation of the segmentation of barrel contours in neighboring sections starting from only one set of manual landmarks placed in a central section, a *Section* object is created for each physical barrel field section. This *Section* object contains information about its vertical position relative to the previous and next physical section, together with a transformation matrix that allows alignment with these sections in the x-y plane. This allows automatically propagating the segmentation results of the first, central section outwards towards the top and bottom barrel field sections. Further, after segmentation of all barrel field sections, the segmented barrel contours are available as aligned 3D stacks. This allows the next step (evaluation and optimization of segmented barrel contours) to proceed fully automated. The user only needs to interact with the final segmented 3D barrel contours, if desired.

Detailed description:

Segmentation of the barrel contours from all optical sections is semi-automated. First, the blood vessel pattern is detected in a median projection image of the z-stack of each tangential section. The blood vessel patterns of adjacent tangential sections are aligned pairwise either manually or automatically and the resulting transformations (translations and rotations) are used during the automatic segmentation to align the segmented barrel contours of these neighboring tangential sections. Landmarks are manually placed in the center of all barrels that are going to be segmented in a central tangential section in which all barrels are visible **(Figure 2A)**.

The segmentation of the barrel contours in a single optical section consists of three steps: gray value-based image filtering, landmark-based region growing and landmark based object segmentation.

*Image Filtering*

The goal of these pre-processing steps is to produce images that clearly separate barrels from their surrounding septa in terms of their gray value range. Fine spatial structures, corresponding to small capillaries and unstained cells, as well as larger blood vessels, are present in the original image, but are considered noise for the purpose of detecting barrels **(Figure 2A, D)**. High spatial frequencies are reduced by a median filter with a radius of 5 pixels to the image. Next, small structures are detected by thresholding a top hat filtered [3] copy of the image. The top hat transform is performed with a binary ball structuring element with a radius of 5 pixels. The threshold T_TH_ is selected as:

$$T_{TH}=\mu_{TH}+0.5\sigma_{TH},$$

where μ_TH_ and σ_TH_ are the mean and standard deviation of the gray values of the top hat image. All pixels with a gray value greater than T_TH_ are labeled as foreground in the initial image by setting them to a gray value of 255. Further smoothing of gray values while preserving the borders between barrels and septa is achieved by applying a bilateral filter [4] with two Gaussian kernels to the image. The width of the spatial kernel is set to 5 pixels, the width of the intensity kernel to 20 (in gray values). This filter leaves the structures labeled as foreground unaffected, because the difference in gray values between the foreground (gray value 255) and barrels and septa is much larger than the width of the intensity kernel of the bilateral filter. The foreground structures are now filled by iteratively replacing pixels on the border of a foreground object by the average gray value of their neighboring non-foreground pixels until no more foreground pixels are present. The last image filtering step is an intensity mapping with a sigmoid filter of the form:

$$I'=\frac{255}{\left(1+\exp\left(-\frac{(I-\beta)}{\alpha}\right)\right)^{2}}.$$

The parameters α and β are computed from the mean μ and standard deviation σ of the image:

$$\alpha=0.4\sigma\wedge\beta=\mu-0.85\sigma.$$

The result of the image filtering pipeline is shown in **Figure 2F** for a single barrel with the surrounding septa. The line profile in **Figure 2E** shows a clear distinction between septum and barrel in terms of gray values as compared to the original image **(Figure 2D)**.

*Landmark-based region growing*

This step turns the filtered gray value image into a binary image, where individual pixels are labeled as belonging to either barrels or background. To compensate for variations in gray value distributions between different areas of the image, this step is performed separately on regions of the image defined by the landmarks placed in the first step **(Figure 2A)**. The regions are defined by the regions of the Voronoi diagram [5] of the landmarks. However, all points in a Voronoi region (VR) with a distance of more than 175 pixels to the landmark are not included in the region. This results in a subdivision of the image into regions that resemble the barrels in their shape **(Figure 2B)**. For each region, an optimal threshold T_opt_ for separating barrel-related and septum-related pixels and a seed threshold T_s_ for the starting seed of the region growing are computed. T_opt_ is computed from the gray value histogram of the region using Otsu’s method [10] **(Figure 2E)**. T_s_ is set to:

$$T_{s}=I_{\min}+\max(\mu-0.5T_{opt},10).$$

Here, I_min_ is the minimum gray value and μ the mean gray value of all pixels in this region. All pixels in this region with a gray value of at most T_s_ are labeled as segmented. Now, the threshold is increased from T_s_ to T_opt_ in steps of 1. During each step, all pixels in this region with gray value equal to the current threshold and at least one pixel labeled as segmented in their 8-neighborhood [1] are also labeled as segmented; otherwise, they are labeled as not segmented. This prevents oversegmentation of the image by requiring that all segmented pixels are connected to the seed regions. The choice of the seed region threshold has been optimized to select pixels that clearly belong to the barrel. Thus, each region can be reliably segmented into barrel- and septum-related pixels independent of overall gray value variations in the image **(Figure 2E-G)**.

*Landmark-based object segmentation*

Next, the binary segmented image is turned into an object image, where every object represents the final segmentation of a barrel **(Figure 2C)**. First, all connected components in the binary image are labeled with a unique identifier. If a connected component is located inside only one VR, it is re-labeled with a unique identifier corresponding to the barrel inside this VR. A connected component may also be present in more than one VR. In this case, it is determined whether it is necessary to split this connected component into several components. A connected component is split along the border between adjacent VR if both of the following criteria are true:

- It consists of at least 10000 pixels.
- The number of pixels in each VR where it is present is more than 10% of its total size; or the number of pixels of this component in a VR is more than 20% of all segmented pixels in this VR.

These criteria ensure that large, falsely connected components are split while small components and small parts of large components that just extend into an adjacent VR are left intact. An erosion with a binary ball of radius 1 followed by a closing with a binary ball of radius 3 are applied. This ensures that split objects are separated and small artifacts are removed. Now all components are re-labeled according to the unique identifier of the barrel inside the VR they are assigned to, in this case the C2 barrel **(Figure 2H)**. In the final step, all labeled components belonging to the same barrel are processed separately from all components belonging to different barrels. The component closest to the landmark position of the corresponding VR is labeled as the central component. All components with a maximum distance of 30 pixels to another component are considered to belong to the same object. In case the components in a VR belong to more than one of these objects, only the object containing the central component is kept and all others deleted. This removes outlying segmentation artifacts. Finally, the remaining components are merged by applying a closing filter with a binary ball of radius 40 and possibly remaining holes in the object are filled **(Figure 2I)**. The contour of this barrel object is extracted as described for the pia and WM contours. Smoothing of the contour vertices is performed by averaging 10 neighboring vertices. Every 10^th^ vertex is then sampled and added to the contour of this barrel in a single optical section **(Figure 2A)**.

After all optical sections of a tangential section have been processed in this way, the barrel landmarks for the adjacent tangential section are automatically determined. For each barrel, the centroids of the five contours of the optical sections closest to the adjacent tangential sections are averaged. The transformations obtained earlier through pairwise alignment of adjacent sections are used to compute the position of this centroid in the next tangential section. Repeating this step for all barrels allows automatic determination of the Voronoi diagram for the next tangential section based on the segmented barrel contours. This guarantees continuity of the segmented barrel outlines between different tangential sections.

*Reconstruction of 3D barrel dimensions*

The 3D structure of a barrel is obtained by segmentation of the barrel contours for all optical sections, ordering these contours in the z direction and alignment of adjacent tangential sections as described above. The vertical extent of the barrel is determined by evaluating and, if necessary, optimizing the segmented contours with respect to a simple geometric model of barrel shape.

Each contour is evaluated on the filtered gray value image after bilateral filtering and without taking pixels labeled as foreground into account **(Figure 3A)**. This effectively amounts to evaluation of the barrel contour on a low-pass filtered version of the original image. For all pixels inside and outside of the contour, the mean of the gray values is calculated from the respective histogram **(Figure 3B)**. The ratio of means:

$$r=\frac{\mu_{o}}{\mu_{i}},$$

where µ_o_ is the mean of all pixels outside of the contour and µ_i_ is the mean of all pixels inside of the contour, is used to measure the quality of barrel segmentation with this contour. A good segmentation result separates the brighter septum from the darker barrel pixels and is therefore expected to have a high value of r. However, for quantitative evaluation independent of experimental variability, it is necessary to compare this number to a reference. For this purpose, the ratio of means is also computed for two circles with different radii centered on the VR of this barrel in each optical section **(Figure 3A)**. By computing an average radius for the segmented contour:

$$R=\sqrt{\frac{A}{\pi},}$$

where A is the number of pixels inside the contour, it is possible to evaluate the segmented contour relative to the reference contours (circles) as a function of the radius **(Figure 3C)**. In this example, the ratio is increasing as a function of the contour radius. The segmented contour has an even higher value than a simple linear extrapolation of the two reference contours to the segmented contour radius. This means, according to this criterion, the segmentation is a good approximation to the actual barrel outline.

Evaluating the ratio of means as a function of the contour radius is also used to optimize the segmented contours according to a geometric model of the barrel shape. If the ratio of means increases with increasing radius of the reference contours, the barrel contour is also expected to be large. In case the ratio of means decreases with increasing radius, the barrel contour is expected to be small. A segmented contour is optimized if the following criteria are met:

- The ratio of means r is smaller than the average of r for all contours
- The slope of the line defined by the ratios of the two reference contours as a function of the radius is smaller than 0.005/pixel
- The ratio of means r is less than 15% above the value of the linear extrapolation from the two reference contours to the radius of the segmented contour

The optimized segmentation also uses the smoothed image after bilateral filtering. Region growing proceeds as described earlier for the initial segmentation within the VR of this barrel, however, the optimal upper threshold T_opt_ is selected differently. The optimal threshold is selected by finding the gray value threshold T for which the following function is maximized:

$$\alpha(T)=r(T)\cdot\frac{A(T)}{N(T)}.$$

Here, N is the number of pixels with gray value lower than T, A is the area of a circle around these pixels and r is the ratio of means of this circle. Qualitative analysis of the behavior of this function in the presence or absence of a barrel in the VR shows how this can be used to optimize oversegmented, large contours to smaller contours with higher values of r. If the barrel is present in the VR, there are spatial correlations between pixels of similar gray values. Increasing from low values, the gray values increase towards the barrel-septum border and become highest in the septum. With increasing threshold T, more pixels are segmented within a larger area. Therefore, it approximately holds that $A(T)\propto N(T)$and maximizing α automatically maximizes r. Conversely, if no barrel outline is present in the VR, there are no spatial correlations between pixels of the same gray value, and therefore *A~const* independent of T. The segmented contour then covers a large part of the VR. This can be used to detect the location of the barrel top and bottom contours. The optimization yields small contours at the barrel top and bottom, followed by an increase in the contour diameter. This is illustrated in **Figure 3D-E**. The side view of the C2 barrel in **Figure 3D** shows all contours after the automatic alignment of the tangential sections and optimized segmentation. The barrel top and bottom contours are automatically found by detecting local minima in the contour diameter followed by a large increase in the contour diameter, in line with the model described above, and are labeled in green for visualization. These minima are detected with an accuracy of about 10µm, because there are usually several adjacent optimized contours with small diameter before the contour diameter increases again. Thus, the overall accuracy in determining the 3D barrel extent is about 15µm. The gray contours represent spurious contours above and below barrel top and bottom. **Figure 3E** demonstrates that the optimization works as described. The top and bottom contours are shown after the regular (red) and optimized (green) segmentation. The slope of the line formed by the two reference contours is negative, in contrast to the contour in the center form the middle of the barrel (see bold red line in **Figure 3D**), where the slope of the reference line is positive. The optimization of the top and bottom contours leads to smaller diameters and an improved quality of the contours, as measured by the ratio of means.

**3D reconstruction of anatomical landmarks *(Supplemental Figure S3)***

Technical note:

The VTK libraries are used for handling of spatial data structures and geometric and surface reconstruction algorithms. The ITK libraries are used for handling of image data structures, image filters and pixel- and pixel-neighborhood iterators to compute the distance transform of 2D contours.

Detailed description:

After the segmentation, all anatomical landmarks are present in the form of 2D contours in the tangential plane defined by the slicing direction. The 3D structure is recovered by ordering contours of tangential and optical sections in the z direction. The barrel contours are then merged with the pia and WM contours by matching the blood vessel pattern in corresponding optical sections. Now, the parameters describing the anatomical landmarks in 3D have to be determined independent of the tangential slicing direction.

First, each barrel is smoothed by removing outliers in single contours and smoothing contours along the slicing direction. A point in a contour is considered an outlier if its distance to the contour centroid is greater than µ + 1.7σ. Here, µ is the mean and σ is the standard deviation of the distances of all points in all contours of a barrel to the axis passing through the barrel centroid along the slicing direction. If a point is found to be an outlier, its distance to the contour centroid is reduced to the average distance of all non-outlier points in the same contour to the contour centroid. Then, the barrel contours are smoothed along the slicing direction. Every contour is sampled at equal angular intervals of 10°. Every sampled point is replaced by the average of points sampled at the same angle in 50 neighboring contours along the slicing direction. This removes artifacts due to slicing of the cortex into 50μm thick sections **(Figure 4A)**.

For reconstruction of the pia and WM as 2D surfaces, a heuristic method is used for extrapolation of the surface at the top. This is necessary because the exact thickness and shape of the first tangential section are unknown. First, the signed 2D distance transform is computed for each contour. Then, the distance transform is iteratively extrapolated to section i = 1, 2, …, n above the first section according to the following equation:

$$d_{i}(x,y)=d_{i-1}(x,y)+\left(d_{i-1}(x,y)-d_{i-2}(x,y)\right)+(i-1)^{2}$$

The first two terms are a linear extrapolation of the distance transform, and the last term introduces a curvature. The surface is now generated by computing the isosurface of the distance transform at value 0 using the Marching Cubes algorithm [7]. A low-pass filter [8] is applied to the resulting surface to remove discontinuities and slicing artifacts.

Cortical blood vessels are reconstructed as lines in 3D and represented by a starting point and an end point. The normalized vector between these two points represents the vessel orientation. Segmented vessels have a diameter between 7µm and 115µm. These vessels usually have an orientation perpendicular to the pia. This feature is used in their automatic reconstruction. Starting in the first section, for each vessel contour the nearest contour in the next section is determined. This search is iteratively repeated in deeper sections in the direction of the vector connecting these two contours. When no more vessel contours can be found in this direction, the reconstructed vessel is replaced by a 3D vector pointing from the deepest vessel contour to the highest vessel contour, i.e. from the WM towards the pia. If the vessel consists of more than 12 contours, all contours deeper than the 12^th^ contour are discarded. This is necessary because large vessels may deviate from a straight line in deep cortex. In addition, each vessel is evaluated for reconstruction quality by calculating the angle between the vessel orientation given by the unit vector $\hat{a}$ and the surface normal $\hat{n}$ at the intersection point between the pia and the extrapolated vessel. If this angle is greater than 10°, the vessel is not used during reconstruction of the BC axis.

In the final step, the orientation of each barrel and the corresponding barrel column in 3D is reconstructed by finding a 3D BC axis. Candidate axes are selected by computing the distance *d* of the barrel centroid to all pia surface triangles within a radius of 2mm, the unit vector $\hat{b}$ in this direction and the surface normal $\hat{n}$ of the corresponding surface triangle. A score:

where $d_{\max}$ is the maximum distance of all surface triangles to the barrel centroid, is assigned to every candidate axis. This score is high for axes that are perpendicular to the pia surface and a short connection between the pia and the barrel center. The BC axis maximizes this score under the constraint that it is oriented similarly to the vessels in and around the barrel. This constraint is implemented by selecting the axis with the highest score and a maximum angle of 5° with the vessel orientation that is closest to the average orientation of all vessels within a radius of 350µm around the barrel centroid. After BC axes have been computed separately for every barrel, possible systematic errors resulting from the limited resolution of the reconstruction of each BC axis are minimized by iteratively removing components of the BC axes that are convergent towards neighboring axes in the direction of the pia.

Now, the barrel top and barrel bottom points are computed by projection of the maximum extent of the top and bottom contours onto the BC axis and, if necessary, interpolating additional contours between these points and the top and bottom contours until the barrel is closed at top and bottom.

The last step in the barrel reconstruction is the calculation of an average barrel contour. This is necessary in order to be able to define the barrel cross-section area and the barrel and barrel column volume in a manner that is consistent with manual tracings of the barrel contours. The average contour is sampled at the same angular intervals of 10° as all other contours. The distance of every sampled point to the BC axis is set to µ + 1.0σ, where µ and σ are the mean and the standard deviation of the distance of all points sampled at the same angle to the BC axis. The parameters are chosen such that the contour resembles the maximum extent of the barrel. In case the average contours of neighboring barrels overlap, the overlapping points are automatically detected and modified to no overlap by setting them to a distance of 10µm apart. This may be necessary because the segmented contours of neighboring barrels do not overlap; any overlap is therefore an artifact of the process creating the average contours.

The barrel columns are obtained by extrapolation of the average contours along the BC axis to the intersection points of the BC axis with the pia and WM surfaces, respectively **(Figure 4B)**.

**References**

1. Gonzalez RC, Woods RE (2002) Digital image processing‎, Prentice Hall, New Jersey, 716p.

2. Oberlaender M, Bruno RM, Sakmann B, Broser PJ (2007) Transmitted light brightfield mosaic microscopy for three-dimensional tracing of single neuron morphology. J Biomed Opt 12: 064029.

3. Soille P (2002) Morphological Image Analysis: Principles and Applications, Springer, Berlin Heidelberg.

4. Tomasi C, Manduchi R (1998) Bilateral filtering for gray and color images. Proceedings of the 1998 IEEE International Conference on Computer Vision, Bombay, India, pp. 839-846.

5. de Berg M, Overmars M, Cheong O, van Kreveld M (2010) Computational Geometry: Algorithms and Applications‎. Springer-Verlag Berlin Heidelberg, 386p.

6. Dercksen VJ, Weber B, Guenther D, Oberlaender M, Prohaska S, et al. (2009) Automatic Alignment of Stacks of Filament Data. IEEE Int Symp on Biomedical Imaging: From Nano to Macro (ISBI) 971-974

7. Lorensen W, Cline HT (1987) Marching cubes: A high resolution 3D surface construction algorithm. ACM Siggraph Computer Graphics 21:4, 163-169.

8. Taubin G, Zhang T, Golub G (1996) Optimal surface smoothing as filter design. Computer Vision—ECCV'96: 283-292.

9. Dijkstra EW (1959) A note on two problems in connexion with graphs. Numerische Mathematik 1: 269-271.

10. Otsu N (1979) A threshold selection method from gray-level histograms. IEEE Trans. Sys., Man., Cyber. 9: 62–66.
